# Anomaly Detection Models for SARS-CoV-2 Surveillance Based on Genome *k*-mers

**DOI:** 10.3390/microorganisms11112773

**Published:** 2023-11-15

**Authors:** Haotian Ren, Yixue Li, Tao Huang

**Affiliations:** 1 Bio-Med Big Data Center, CAS Key Laboratory of Computational Biology, Shanghai Institute of Nutrition and Health, University of Chinese Academy of Sciences, Chinese Academy of Sciences, Shanghai 200031, China; renhaotian2021@sibs.ac.cn; 2 Key Laboratory of Systems Health Science of Zhejiang Province, School of Life Science, Hangzhou Institute for Advanced Study, University of Chinese Academy of Sciences, Hangzhou 310024, China; 3 Guangzhou Laboratory, Guangzhou 510005, China; 4 School of Life Sciences and Biotechnology, Shanghai Jiao Tong University, Shanghai 200240, China; 5 Collaborative Innovation Center for Genetics and Development, Fudan University, Shanghai 200433, China

**Keywords:** anomaly detection, virus surveillance, SARS-CoV-2, *k*-mer, machine learning

## Abstract

Since COVID-19 has brought great challenges to global public health governance, developing methods that track the evolution of the virus over the course of an epidemic or pandemic is useful for public health. This paper uses anomaly detection models to analyze SARS-CoV-2 virus genome *k*-mers to predict possible new critical variants in the collected samples. We used the sample data from Argentina, China and Portugal obtained from the Global Initiative on Sharing All Influenza Data (GISAID) to conduct multiple rounds of evaluation on several anomaly detection models, to verify the feasibility of this virus early warning and surveillance idea and find appropriate anomaly detection models for actual epidemic surveillance. Through multiple rounds of model testing, we found that the LUNAR (learnable unified neighborhood-based anomaly ranking) and LUNAR+LUNAR stacking model performed well in new critical variants detection. The results of simulated dynamic detection validate the feasibility of this approach, which can help efficiently monitor samples in local areas.

## 1. Introduction

Ever since the COVID-19 outbreak, the SARS-CoV-2 virus has undergone numerous mutations, leading to the emergence of different variants [1]. Towards the end of 2020, the World Health Organization (WHO) classified certain variants as variants of interest (VOI) and variants of concern (VOC) due to their significant impact on the transmission, severity and effectiveness of vaccines and prevention strategies [2]. One notable variant is the Omicron variant, whose parent lineage was listed as a VOC by the WHO on 6 November 2021, which did not end until 14 March 2023. In addition, the subvariants of Omicron, XBB.1.5 and XBB.1.16, were defined as VOI on 11 January 2023 and 17 April 2023, respectively, and both remain VOI as of 6 November 2023 [2,3,4].

It is crucial to swiftly identify samples of new virus variants that pose challenges to epidemic prevention and control. By detecting them in a timely manner, we can promptly implement appropriate measures, such as isolation and treatment, to effectively address new virus outbreaks in the region.

In China, nasal and throat swabs are predominantly used for collecting human virus samples. Additional techniques like saliva sampling are also employed [5]. These samples are then subjected to genome sequencing technologies to obtain the genetic code of the virus variants [6,7]. This is just the beginning of understanding this virus variant. The virus genome sequence is usually utilized for phylogenetic analysis, which helps researchers gain more insights into the virus lineage, expression proteins and mutation sites. This valuable information aids in further studying the various features of the virus [8,9]. Sequence alignment [10] plays a significant role in the phylogenetic workflow. However, due to the vast number of COVID-19 sequence samples and the large size of the SARS-CoV-2 virus genome (approximately 30 kb) [11], performing multiple sequence alignment [10,12] becomes computationally complex and time-consuming [13]. This impedes the swift detection of variants. Despite the existence of excellent multiple sequence alignment tools like UShER [14] for SARS-CoV-2, they still do not meet the speed requirements for frontline workers.

Therefore, our approach involves utilizing an alignment-free method [13] that relies on the numerical properties of sequences to compare them. This allows us to bypass issues associated with sequence alignment. One widely used method in this category is *k*-mer analysis [15,16,17]. A common step is to break a reference sequence into *k*-mers and use them to create a hash table. Then, target sequences are broken into *k*-mers and queried against the hash table to check for shared *k*-mers [18]. Several tools, such as VirFinder [19], CAFE [20], kmer2vec [21] and KINN [22], are built upon the concept of *k*-mer analysis. Various *k*-mer models have been developed to optimize sequence analysis and comparison. For example, Jia Wen et al. [23] proposed a *k*-mer sparse matrix model for sequence comparison. Furthermore, rather than utilizing phylogenetic methods focused on virus evolution and structural characteristics, we have opted for an anomaly detection algorithm widely employed in diverse industries [24]. By employing machine learning techniques, our intention is to swiftly identify noteworthy new variants from a substantial collection of virus sequence samples.

Anomaly detection is an important research direction in the field of machine learning, whose purpose is to identify “outliers” in data, as the name suggests. Anomaly detection can be further classified into outlier detection [25] and novelty detection [26], which have certain conceptual differences. The former refers to determining whether a certain data point is abnormal in the case of known data distribution, while the latter refers to finding novel data points different from the existing data in unknown datasets [27,28]. In our case, we aim to detect the emergence of VOC in a specific region during a certain period, aligning with the principles of novelty detection. To accomplish this, we have selected six different types of anomaly detection models from the PyOD [29] toolkit to conduct a variety of test evaluations on this detection task, which are as follows: empirical cumulative distribution based outlier detection (ECOD) [30], one-class support vector machines (OCSVM) [31], k nearest neighbors (KNN) [32], isolation forest (IForest) [33], AutoEncoder [34] and learnable unified neighborhood-based anomaly ranking (LUNAR) [35]. Additionally, we propose a method for stacking models to make predictions for new critical variants. Stacking is an ensemble learning technique where multiple base learners are combined with a meta learner, using the base learners’ outputs as input to the meta learner [36]. In these prediction tasks, the models will train the known “normal samples” to determine whether there are “abnormal samples” in the new dataset, which is actually semi-supervised learning [37].

Giovanna Nicora et al. [38] also had a similar idea, but they used one class SVM to analyze the spike protein sequence of SARS-CoV-2. However, this method still requires the support of phylogenetic techniques, making the data processing more complex in practical applications. Additionally, this approach may not be suitable for situations where a specific virus variant remains dominant in a particular region over time. Because, even if the virus variant is VOC/VOI, it is just a “normal” circulating strain, since it has been popular in the region during this period. Hence, identifying the strain as “abnormal” in such cases lacks practical significance.

**Figure 1:**
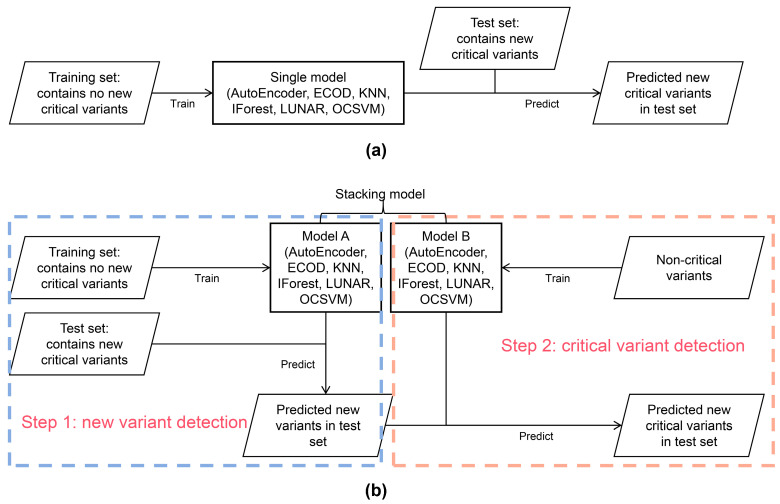
An overview of the models used to detect new critical variants in this work. (**a**) Detect new critical variants using a single outlier detection model; (**b**) detect new critical variants using the stacking model.

Based on the above considerations, this study aims to detect new critical variants that may appear in the samples collected over a period. We evaluate the efficacy of different anomaly detection algorithms on the *k*-mers of the SARS-CoV-2 genome to determine a suitable model for real-world epidemic surveillance. In this work, we consider variants that were once defined as VOC as critical variants, and the variants that were never defined as VOC are not critical variants. The reason why a VOC is only used as a critical variant is that the public health impact of a VOI is significantly smaller than that of a VOC, and many VOIs turned out to be benign. We selected six independent anomaly detection models. At the same time, we proposed that these independent models could be stacked to complete the prediction task of new variant detection and critical variant detection, so as to improve the interpretability of the prediction process of new critical variant detection (Figure 1). We tested and evaluated these models in various ways using public datasets and simulated their use in real world scenarios.

## 2. Materials and Methods

### 2.1. Data Source

From the EpiCoV database, which is a repository of information on SARS-CoV-2 in the Global Initiative on Sharing All Influenza Data (GISAID) [39], we obtained the sample sequences of complete SARS-CoV-2 genome sequences from human hosts and their metadata in Argentina, China and Portugal between 2020 and 2022. These countries, situated on different continents, have each implemented national epidemic control measures, although the timing and intensity of these policies varied. China gradually liberalized epidemic control until the end of 2022 [40]. Portugal was cited as a COVID-19 success story due to the low number of deaths in the early stages of the pandemic [41]. Argentina also implemented strict nationwide lockdown measures in phases during the year 2020 [42]. Moreover, all three countries achieved high rates of vaccinations [43]. We selected samples of these three countries as model test data to simulate the implementation of new critical variant anomaly detection under reasonable and effective epidemic prevention and control policies, so as to evaluate the feasibility of this surveillance approach.

### 2.2. Data Processing

**Figure 2:**
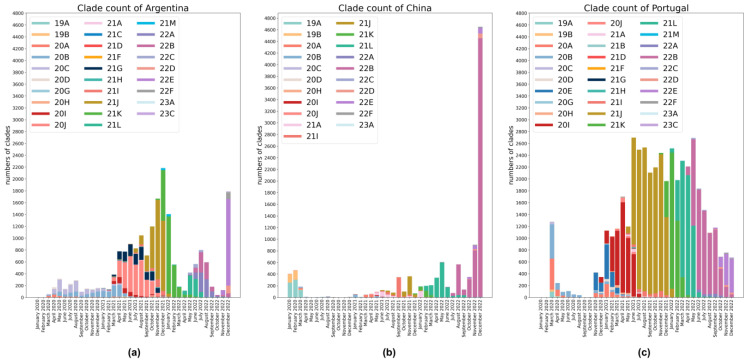
An overview of complete SARS-CoV-2 genome sequences in human hosts from three countries in the years from 2020 to 2022. (**a**) Statistics of SARS-CoV-2 variants in Argentina; (**b**) statistics of SARS-CoV-2 variants in China; (**c**) statistics of SARS-CoV-2 variants in Portugal.

We input the sequence file into the pipeline of Nextclade [44] to obtain the table file including the Nextstrain lineage information for each sample. Next, we calculated the *k*-mers for all sequences. A set of subsequences of length k in a biological sequence is called *k*-mer, and a sequence of length N has N–k+1 *k*-mers. RNA sequence contains 4 ribonucleotides, so there are up to 4 k types of *k*-mers. To control the number of *k*-mers and ensure that valid sequence characteristic information can be retained, we set k to 5, so there were 1024 *k*-mers. The table of each sequence *k*-mers was combined with its sample name, serial number, collection time, lineage and other information. And the samples with incomplete information were filtered out. In the end, we obtained a total of 74,885 sample data, including 9485 sequence samples that were consistently excluded from VOC. The sample statistics of each country are shown in Figure 2.

### 2.3. Anomaly Detection Models

There are many types of existing anomaly detection models. According to the classification of individual anomaly detection models by PyOD [29], we selected six different types of anomaly detection models for research, which are as follows: ECOD [30] based on probability, OCSVM [31] based on linear model, KNN [32] based on proximity, IForest [33] based on outlier ensembles, AutoEncoder [34] based on neural networks and LUNAR [35] based on graphs. We used these six anomaly detection models to simulate the monitoring of samples from three countries to explore the potential of these models in the detection of new critical variants. At the same time, to improve the interpretability of the anomaly detection process, we have divided the task of new critical variant detection into two steps, namely, new variant detection and critical variant detection. We combined these six anomaly detection models to match two different steps, resulting in a total of 36 stacking models. We describe the stacking model in the form of “model A + model B”, where model A is the model used in the new variant detection and model B is the model used in the critical variant detection. We conducted a comprehensive evaluation of the performance of these single models and stacking models on the task of detecting new critical variants. To be specific, it includes the following tasks: model evaluation of new variant detection; model evaluation of critical variant detection; model evaluation of new critical variant detection; comparison of the ability of these models to detect all critical variants on the day they first appear in the three countries; and analog dynamic monitoring. The source code of our work is available at https://github.com/sweety919/Anomaly-detection-models-for-SARS-CoV-2-surveillance-based-on-genome-k-mers (accessed on 12 November 2023).

### 2.4. Dataset Preparation

#### 2.4.1. Datasets for Model Evaluation of New Variant Detection

**Figure 3:**
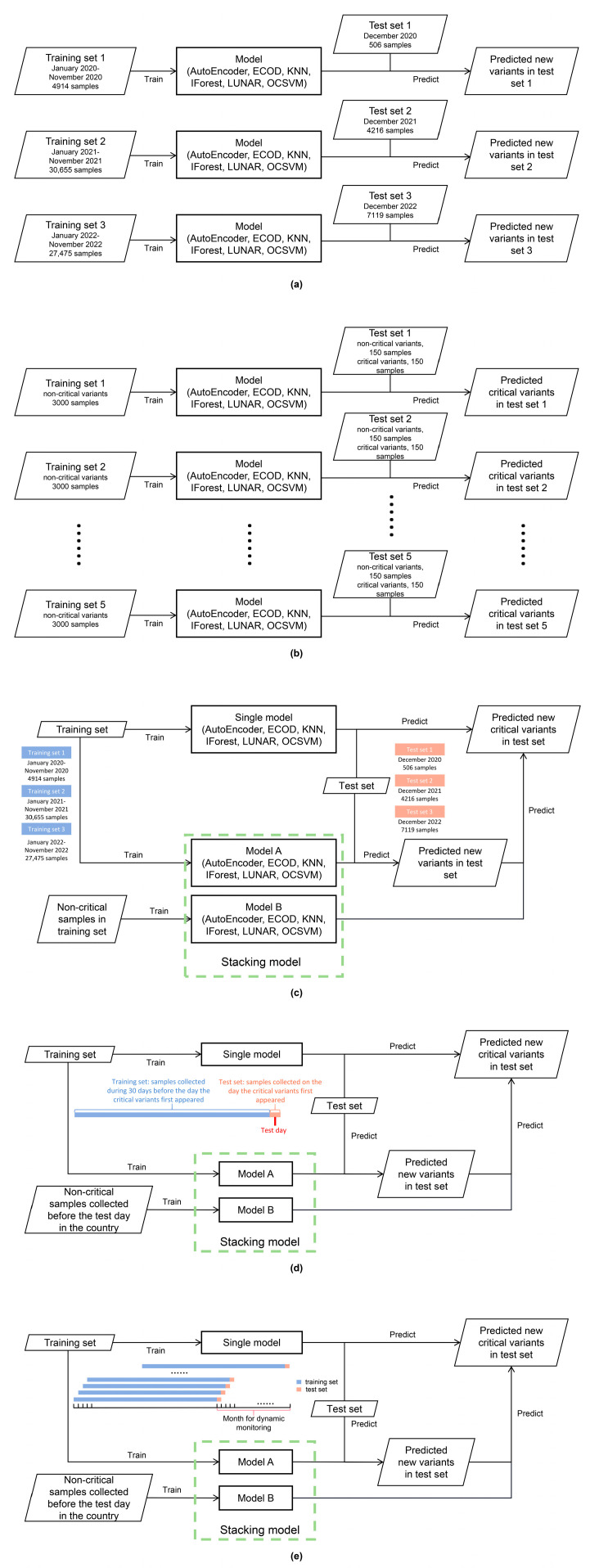
Five rounds of evaluation of anomaly detection models for SARS-CoV-2 surveillance based on genome *k*-mers. (**a**) Evaluation of new variant detection; (**b**) evaluation of critical variant detection; (**c**) evaluation of new critical variant detection; (**d**) compare the detection of all critical variants on the days they first appeared in three countries; (**e**) analog dynamic monitoring.

To evaluate the performance of various anomaly detection models for identifying new variants, we conducted experiments using three distinct datasets (Figure 3a). The first dataset consists of a training set of 4914 samples in three countries from January 2020 to November 2020 and a test set of 506 samples in three countries from December 2020. The second dataset consists of a training set of 30,655 samples in three countries from January 2021 to November 2021 and a test set of 4216 samples in three countries from December 2021. The third dataset consists of a training set of 27,475 samples in three countries from January 2022 to November 2022 and a test set of 7119 samples in three countries from December 2022. During these tests, the training set exclusively comprised “normal samples”, representing variants that have previously occurred. Conversely, any variants present in the test set that were not observed in the training set were classified as “abnormal samples”.

#### 2.4.2. Datasets for Model Evaluation of Critical Variant Detection

To test the ability of different anomaly detection models in the step of critical variant detection (Figure 3b), we conducted a random sampling process from a dataset containing 74,885 samples. From this sampling, we obtained 3000 sequence samples (“normal samples”) that always did not belong to VOC as the training set. In other words, the samples in the training set are not critical variants. At the same time, 150 different sequence samples which are not critical variants (“normal samples”) and 150 VOC sequence samples which are critical variants (“abnormal samples”) were selected to form a test set. This process was repeated five times to create five different datasets.

#### 2.4.3. Datasets for Model Evaluation of New Critical Variant Detection

This test step (Figure 3c) used the datasets which were used in the model evaluation of new variant detection, that is, a total of three datasets. When evaluating a single model, the new critical variant in the test set was considered an abnormal sample. However, when evaluating stacking models, we needed to define abnormal and normal samples in multiple steps. In the first step, samples in the test set that were not included in the training set were considered abnormal samples. Then, the samples that were classified as abnormal in this step were used as the test set in the second step, which aimed to detect critical variants. The training set in this step consisted of all non-VOC sequence samples originally input into the entire stacking model. The samples that were identified as abnormal in the final output represented the model’s decision on new critical variants.

#### 2.4.4. Datasets for Comparing the Detection of All Critical Variants on the Days They First Appeared in Three Countries

In our research, we focused on analyzing VOC in Argentina, China and Portugal from 2020 to 2022. We took all samples collected in the country on the day these critical variants appeared as the test sets (Figure 3d). These critical variants were the real abnormal samples in the test sets. All samples collected in the country during the 30 days before this day were training sets, and all non-VOC occurring in the country before this day were training sets for the critical variant detection step in the stacking models.

#### 2.4.5. Datasets for Analog Dynamic Monitoring

To simulate dynamic monitoring, we selected one month from different time periods for each of the three countries (Figure 3e). For each selected day, the samples collected in that country formed the test set. The corresponding training set for that day consisted of all the samples collected in that country during the previous 30 days. Similarly, any non-VOC samples present in the country prior to that day were included as training sets for the critical variant detection step in the stacking models.

## 3. Results

### 3.1. Evaluation of New Variant Detection

In this section, we conducted performance testing on six anomaly detection models using three different datasets. All models utilized the default parameters provided by PyOD [29]. We evaluated the models using various metrics, such as the Matthews correlation coefficient (MCC) [45], f1-score, accuracy, recall, accuracy, specificity and area under the curve (AUC). The average values of these metrics were calculated (Table 1 and Table S1). The recall metric indicates the model’s ability to identify correct “abnormal samples”, while the AUC reflects the model’s classification performance. The MCC is a comprehensive indicator that assesses the model’s classification ability in the presence of imbalanced sample categories. Thus, our focus was on these three metrics. From our analysis, we observed that both KNN and LUNAR models outperformed other models in terms of their capability to detect “abnormal samples” accurately. However, it is worth noting that most models struggled with the MCC, particularly those with MCC values below 0, indicating poorer performance in classifying imbalanced datasets.

### 3.2. Evaluation of Critical Variant Detection

We also tested the ability of the six models to detect critical variants. We calculated the evaluation metrics of the models’ predictions over five prepared datasets and then took their average (Table 2 and Table S2). Compared with the poor performance of many models in the step of new variant detection, the prediction effect of most models in critical variant detection appears to be quite good, because these models have learned the non-VOC sequence features well in the training set. This indicates that anomaly detection methods hold significant potential for detecting critical variants. Notably, the AutoEncoder and LUNAR models, both of which are anomaly detection methods based on deep learning, exhibited superior performance. This highlights the advantages of deep learning methods in handling complex relationships and features within sequences. We know that *k*-mers carry sequence information. Each *k*-mer does not exist alone, and there may be complex correlations among them which are also very likely to contain sequence characteristics. While conventional machine learning methods struggle to handle such high-dimensional information, deep learning methods can extract crucial features that contribute to excellent classification abilities.

### 3.3. Evaluation of New Critical Variant Detection

In this round of evaluation, we tested the ability of 6 single models and 36 stacking models to detect new critical variants, calculated the metrics and averaged them (Table 3 and Table S3). We found that the effects of KNN, LUNAR and three stacking models, which are KNN+KNN, KNN+LUNAR and LUNAR+LUNAR, were relatively outstanding among all models, with recall rates exceeding 0.5 and AUC scores surpassing 0.6. However, our findings also indicate that, similar to the evaluation outcomes for new variant detection, the low MCC values highlight room for improvement in the models’ classification abilities, particularly when dealing with imbalanced datasets.

### 3.4. Comparing the Detection of All Critical Variants on the Days They First Appeared in Three Countries

**Figure 4:**
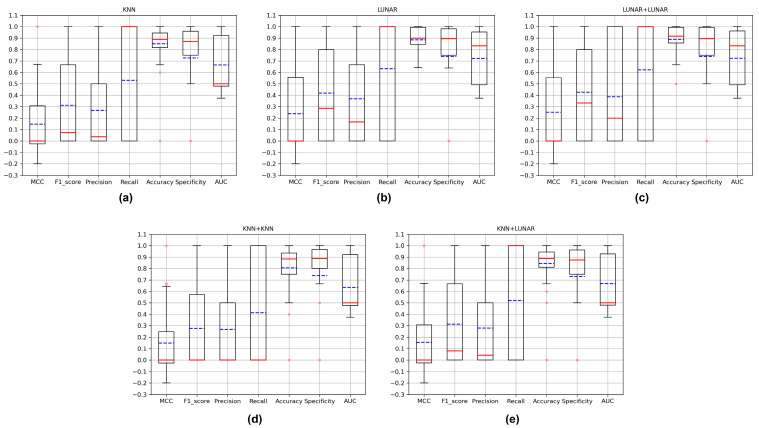
Compare the detection of all critical variants on the days they first appeared in three countries. (**a**) The evaluation metrics of KNN; (**b**) the evaluation metrics of LUNAR; (**c**) the evaluation metrics of LUNAR+LUNAR; (**d**) the evaluation metrics of KNN+KNN; (**e**) the evaluation metrics of KNN+LUNAR. The red lines in the boxplots indicate the medians, the blue lines indicate the mean values and the pink dots indicate the outliers.

**Figure 5:**
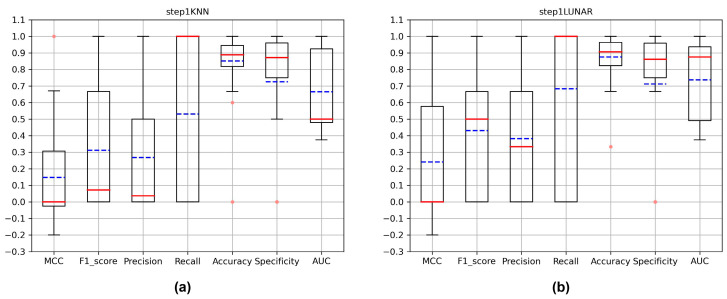
Compare the new variant detection of all critical variants on the days they first appeared in three countries. (**a**) The evaluation metrics of KNN; (**b**) the evaluation metrics of LUNAR. The red lines in the boxplots indicate the medians, the blue lines indicate the mean values and the pink dots indicate the outliers.

For all VOCs that occurred in Argentina, China and Portugal between 2020 and 2022, we investigated the days they first appeared in each country. And we calculated the number of samples collected on the days the critical variants first appeared, as well as the samples collected in the 30 days before the critical variants appeared (Table S4). After a preliminary model evaluation of the new critical variant detection, we found that KNN, LUNAR, KNN+KNN, KNN+LUNAR and LUNAR+LUNAR performed better than other models, so we used these models for further testing. They were used to predict samples collected on the day all critical variants first appeared in the three countries. Although the training set entered by the stacking model in the new variant detection is the samples collected in the 30 days before the critical variant appeared, the training set used in the critical variant detection is all the non-VOC samples recorded in this country before the day when the critical variant appeared. We calculated the MCC, f1-score, accuracy, recall, accuracy, specificity and AUC for each round of testing, then compared and counted them (Figure 4). Since on the day that each new critical variant appeared, and usually only the variant sample was an anomaly, this accounts for a small proportion of the test set. So, this is a very unbalanced data set, and this is the reason why the models performed poorly on the MCC evaluation index. We believe that, if the MCC is greater than 0, the classification ability of the model is stronger than that of random classification. As shown in Figure 4, the median of the MCC is around 0, while the average is greater than 0. Combined with the distribution of the AUC, these models have a certain ability to detect new critical variants. In addition to the MCC and AUC, we also paid special attention to the recall. According to Table S4, we can see that the number of new critical variants in the test sets is mostly 1, which caused the model to display a recall of 0 when the critical variants were not correctly identified, and a recall of 1 when they were identified. This explains why the distribution of recall in Figure 4 is from 0 to 1. In this case, though, we can look at the median and average of the recall to compare the performance of the different models. As you can see from Figure 4, LUNAR and LUNAR+LUNAR performed better than the other models. What is more, we further compared the performance of the three stacking models in the new variant detection step. In this step, only KNN and LUNAR play a role. We found that LUNAR was superior to KNN in this step (Figure 5). At the same time, comparing the results of the new variant detection step (Figure 5) with the results of the stacking models after two steps (Figure 4), in terms of the stacking model, the new variant detection step has a great impact on the overall new critical variant detection. And the effect of LUNAR in the first step is better than that of KNN.

### 3.5. Analog Dynamic Monitoring

**Figure 6:**
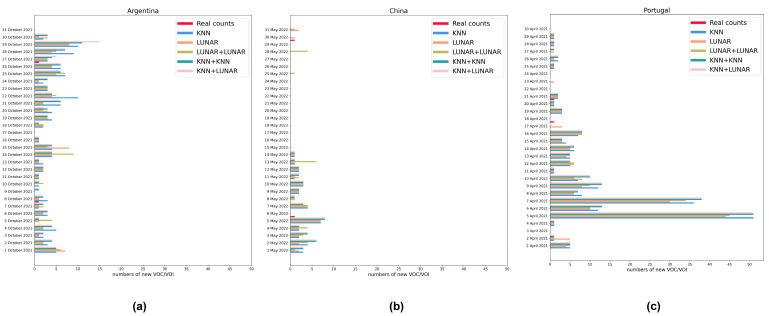
Analog dynamic monitoring of the new critical variants in three countries during a certain period. (**a**) Comparison of the predicted quantity with the actual quantity in Argentina; (**b**) comparison of the predicted quantity with the actual quantity in China; (**c**) comparison of the predicted quantity with the actual quantity in Portugal.

In addition to comparing the detection capabilities of models on the day the critical variants first appeared in the three countries, we also used these five relatively reliable models to simulate real dynamic detection scenarios. We selected a month in different time periods for the three countries and analyzed the samples with the models every day. We compared the number of new critical variants predicted by the models with the actual number of new critical variants (Figure 6). In fact, the days that a new critical variant appeared were a few, and so were the new critical variants. And our models, despite their ability to spot critical new variants on the day they appear, still produced false positives most of the time (Table S5). We used the specificity in Table S5 to calculate the false positive rate (FPR) (FPR = 1 − specificity). According to the bootstrap interval estimation [46], we calculated the 95% confidence interval of the false positive rate of each model: KNN was (0.072, 0.142), LUNAR was (0.100, 0.187), KNN+LUNAR was (0.075, 0.146), KNN+KNN was (0.076, 0.145) and LUNAR+LUNAR was (0.068, 0.136). Summing over all three countries, there were six days on which a new VOC occurred. On three of those six days, all five models predicted at least one new critical variant, but on only two days did all five models correctly predict the real new critical variant. And all five models failed to predict any new variant on two of these six days. On one of these six days, LUNAR correctly predicted one new variant, while the other models did not. Of the 81 days in which no new critical variant appeared, only on 19 of these days did all five models predict no new critical variants. Therefore, at least in the context evaluated, this approach would need to be considerably improved before deployment in a real-world situation. This is because the existing models still have many shortcomings in the case of conditional anomaly detection.

**Figure 7:**
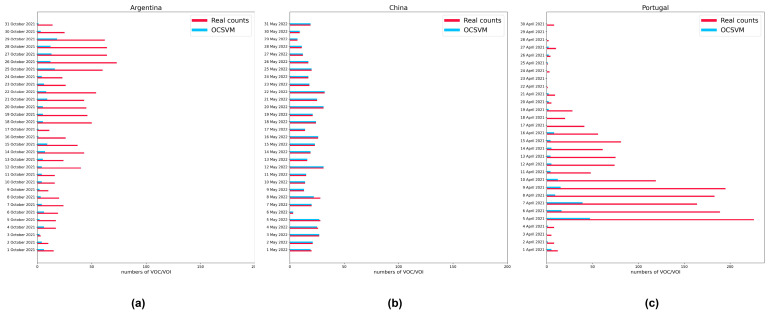
Analog dynamic monitoring of the critical variants in three countries during a certain period using OCSVM. (**a**) Comparison of the predicted quantity with the actual quantity in Argentina; (**b**) comparison of the predicted quantity with the actual quantity in China; (**c**) comparison of the predicted quantity with the actual quantity in Portugal.

When we tested the method of Giovanna Nicora et al. [38] on the data sets used here, we found that their model had much better precision, although there were cases where some VOCs were not identified (Figure 7). This is because the method of Giovanna Nicora et al. [38] was used for VOC detection. For recurring VOCs over a period, the model also considered them abnormal samples. This anomaly detection method is based on the difference of sequence features between VOC and non-VOC. According to the evaluation results of the ability of each anomaly detection model in the critical variant detection, this is reasonable, since many anomaly detection models are fully capable of doing so.

## 4. Discussion

For infectious viruses such as SARS-CoV-2, which are highly transmissible and mutate frequently [47], it is important to detect new and noteworthy variants in a region in good time. These variants may have increased transmissibility and pathogenicity, posing a significant threat to global or regional public health security. Therefore, detecting these variants promptly can assist relevant agencies in rapidly developing prevention and control strategies. We proposed the use of the anomaly detection models to analyze SARS-CoV-2 virus genome *k*-mers and predict the new critical variants that may exist in the collected samples. Multiple rounds of testing and evaluation were conducted on several anomaly detection models, aiming to assess the feasibility of this early warning concept and identify suitable models for real-life epidemic surveillance.

For the performance evaluation of anomaly detection models in detecting new critical variants, we carried out five tests using sample sequences obtained from Argentina, China and Portugal between 2020 and 2022, sourced from GISAID. Throughout the testing rounds, which included new variant detection, critical variant detection and new critical variant detection, we observed that the comprehensive performance of the five models (KNN, LUNAR, KNN+LUNAR, LUNAR+LUNAR and KNN+KNN) surpassed that of the other 4 single models and 33 stacking models examined in this study. Additionally, indicators such as the MCC and AUC demonstrated the models’ capacity to classify samples, even when the categories were highly imbalanced. Subsequently, we employed these five models to assess their ability to detect variants on the day when all critical variants first appeared in the aforementioned three countries. Based on the test results, we have determined that the new variant detection step is crucial in the overall identification of new critical variants for the stacking model. Additionally, LUNAR, as a deep learning method, outperformed KNN in both the independent detection of new critical variants and prediction as part of the stacking models. This demonstrates the significant advantages of LUNAR, which falls under the graph neural network method, in handling complex relationships between features. To assess the feasibility of our approach in real-time epidemic surveillance, we utilized these five models to predict daily samples from three different countries during various periods. Apart from evaluating VOC as a crucial variant, we also conducted tests on both VOC and VOI as crucial variants (Tables S6–S9, Figures S1–S4). Although the models all have certain false positive rates, we pay more attention to the recall of the models because, in virus surveillance, we are more worried about missing abnormal samples. The test results further confirm that LUNAR exhibits the highest level of comprehensive performance among all the models tested across multiple rounds. We compared the performance of our proposed method to the method proposed by Giovanna Nicora et al. [38] on the same data. The results revealed that our method, unlike the method of Giovanna Nicora et al. [38], which solely predicts VOC in samples, incorporates the ability to detect new variants, enabling the identification of new critical variants. This helps reduce the workload of personnel involved in inspecting “key” samples and enhances the efficiency of epidemic prevention and control. However, the detection capability of the five models currently used still has some room for improvement. Therefore, phylogenetic approaches continue to play a crucial role in virus surveillance and early warning. Laboratories equipped with high-performance computing and programming resources may benefit from utilizing analysis pipelines that incorporate phylogenetic considerations.

We propose using anomaly detection models to analyze SARS-CoV-2 virus genome *k*-mers and predict new critical variants that may exist in collected samples, and evaluate some models in various aspects. This approach could be extended to other infectious viruses, such as seasonal influenza viruses [48]. The study in this paper is an attempt to apply machine learning to epidemic surveillance. Despite the limitations of the models tested in this paper, it demonstrates the feasibility of using anomaly detection in epidemic surveillance, even when dealing with large volumes of unanalyzed genomic data. In the future, we have the option to optimize the feature extraction of a viral genome. We observe that a current package, named MathFeature [49], integrates the methods for deriving numerical data from biological sequences. The performance of downstream model predictions may be enhanced by the introduction of these efficient feature extraction methods. Furthermore, we can introduce incremental learning to enable the model to quickly detect real-time data [50], thereby improving its practicality in real-world scenarios.

## 5. Conclusions

This work proposed using anomaly detection models to analyze SARS-CoV-2 virus genome *k*-mers and predict new critical variants that may exist in collected samples. Several anomaly detection models were evaluated through multiple rounds of tests. To verify the feasibility of this virus early warning idea and find a suitable anomaly detection model for actual epidemic surveillance, the dynamic monitoring of SARS-CoV-2 in a real-world scenario was simulated in this work.

## Figures and Tables

**Table 1:** Evaluation of new variant detection.

Model	MCC	F1-Score	Precision	Recall	Accuracy	Specificity	AUC
AutoEncoder [34]	−0.016	0.004	0.002	0.078	0.838	0.842	0.460
ECOD [30]	−0.005	0.008	0.004	0.078	0.887	0.891	0.485
IForest [33]	−0.012	0.005	0.003	0.078	0.857	0.860	0.469
KNN [32]	0.092	0.035	0.018	0.741	0.793	0.792	0.767
LUNAR [35]	0.080	0.036	0.019	0.556	0.837	0.838	0.697
OCSVM [31]	0.006	0.010	0.005	0.207	0.874	0.878	0.543

**Table 2:** Evaluation of critical variant detection.

Model	MCC	F1-Score	Precision	Recall	Accuracy	Specificity	AUC
AutoEncoder [34]	0.539	0.722	0.855	0.625	0.759	0.893	0.759
ECOD [30]	0.122	0.303	0.643	0.200	0.544	0.889	0.544
IForest [33]	0.192	0.372	0.697	0.257	0.576	0.895	0.576
KNN [32]	0.171	0.345	0.691	0.231	0.564	0.897	0.564
LUNAR [35]	0.663	0.811	0.891	0.745	0.827	0.908	0.827
OCSVM [31]	−0.009	0.174	0.487	0.107	0.497	0.888	0.497

**Table 3:** Evaluation of new critical variant detection.

Model	MCC	F1-Score	Precision	Recall	Accuracy	Specificity	AUC
AutoEncoder [34]	−0.014	0.004	0.002	0.078	0.839	0.842	0.460
ECOD [30]	−0.003	0.008	0.004	0.078	0.888	0.891	0.485
IForest [33]	−0.008	0.006	0.003	0.098	0.850	0.853	0.476
KNN [32]	0.105	0.035	0.018	0.852	0.794	0.793	0.822
LUNAR [35]	0.104	0.046	0.024	0.722	0.811	0.811	0.767
OCSVM [31]	0.008	0.010	0.005	0.207	0.875	0.878	0.543
AutoEncoder+AutoEncoder	−0.011	0.004	0.002	0.078	0.860	0.862	0.470
AutoEncoder+ECOD	−0.016	0.001	0.001	0.010	0.920	0.924	0.467
AutoEncoder+IForest	−0.005	0.006	0.003	0.078	0.890	0.893	0.486
AutoEncoder+KNN	−0.010	0.004	0.002	0.078	0.864	0.867	0.473
AutoEncoder+LUNAR	−0.011	0.004	0.002	0.078	0.861	0.864	0.471
AutoEncoder+OCSVM	−0.006	0.007	0.004	0.069	0.887	0.891	0.480
ECOD+AutoEncoder	0.000	0.008	0.004	0.078	0.907	0.910	0.494
ECOD+ECOD	−0.013	0.003	0.002	0.020	0.919	0.923	0.471
ECOD+IForest	0.000	0.008	0.004	0.078	0.906	0.909	0.494
ECOD+KNN	0.000	0.008	0.004	0.078	0.908	0.911	0.495
ECOD+LUNAR	0.000	0.008	0.004	0.078	0.907	0.910	0.494
ECOD+OCSVM	−0.005	0.007	0.004	0.069	0.894	0.897	0.483
IForest+AutoEncoder	−0.002	0.007	0.004	0.078	0.902	0.905	0.492
IForest+ECOD	−0.015	0.001	0.001	0.010	0.921	0.925	0.467
IForest+IForest	−0.003	0.007	0.004	0.069	0.905	0.909	0.489
IForest+KNN	−0.004	0.007	0.003	0.098	0.878	0.881	0.489
IForest+LUNAR	0.000	0.008	0.004	0.088	0.900	0.903	0.496
IForest+OCSVM	−0.006	0.007	0.004	0.069	0.888	0.891	0.480
KNN+AutoEncoder	−0.002	0.007	0.004	0.108	0.876	0.879	0.493
KNN+ECOD	−0.016	0.001	0.001	0.010	0.920	0.924	0.467
KNN+IForest	−0.006	0.006	0.003	0.078	0.888	0.891	0.485
KNN+KNN	0.081	0.031	0.016	0.578	0.864	0.864	0.721
KNN+LUNAR	0.064	0.025	0.013	0.500	0.845	0.845	0.672
KNN+OCSVM	0.011	0.010	0.005	0.254	0.861	0.864	0.559
LUNAR+AutoEncoder	0.008	0.012	0.006	0.108	0.907	0.910	0.509
LUNAR+ECOD	−0.010	0.005	0.002	0.029	0.918	0.921	0.475
LUNAR+IForest	−0.004	0.006	0.003	0.069	0.901	0.904	0.486
LUNAR+KNN	0.075	0.037	0.020	0.412	0.909	0.910	0.661
LUNAR+LUNAR	0.110	0.047	0.025	0.637	0.891	0.891	0.764
LUNAR+OCSVM	0.012	0.011	0.006	0.291	0.834	0.837	0.564
OCSVM+AutoEncoder	−0.005	0.006	0.003	0.059	0.909	0.913	0.486
OCSVM+ECOD	−0.013	0.003	0.002	0.020	0.921	0.925	0.472
OCSVM+IForest	−0.005	0.006	0.003	0.059	0.910	0.913	0.486
OCSVM+KNN	−0.005	0.006	0.003	0.059	0.911	0.914	0.486
OCSVM+LUNAR	−0.005	0.006	0.003	0.059	0.910	0.913	0.486
OCSVM+OCSVM	0.008	0.010	0.005	0.207	0.875	0.878	0.543

## Data Availability

All scripts are available at https://github.com/sweety919/Anomaly-detection-models-for-SARS-CoV-2-surveillance-based-on-genome-k-mers (accessed on 12 November 2023). The data are available in the EpiCoV and GISAID databases.

## Supplementary Materials

The following supporting information can be downloaded at: https://www.mdpi.com/article/10.3390/microorganisms11112773/s1. Table S1: Evaluation metrics of new variant detection; Table S2: Evaluation metrics of critical variant detection: Table S3: Evaluation metrics of new critical variant detection; Table S4: Evaluation metrics of the detection of all critical variants on the days they first appeared in three countries; Table S5: Results of analog dynamic monitoring of new critical variant detection; Table S6: Evaluation of new variant detection (consider VOC/VOI as critical variants); Table S7: Evaluation of critical variant detection (consider VOC/VOI as critical variants); Table S8: Evaluation of new critical variant detection (consider VOC/VOI as critical variants); Table S9: Results of analog dynamic monitoring of new critical variant detection (consider VOC/VOI as critical variants); Figure S1: Compare the detection of all critical variants on the days they first appeared in three countries (consider VOC/VOI as critical variants); Figure S2: Compare the new variant detection of all critical variants on the days they first appeared in three countries (consider VOC/VOI as critical variants); Figure S3: Analog dynamic monitoring of the new critical variants in three countries during a certain period (consider VOC/VOI as critical variants); Figure S4: Analog dynamic monitoring of the critical variants in three countries during a certain period using OCSVM (consider VOC/VOI as critical variants).
