# Supplementary material for: Anomaly Detection Models for SARS-CoV-2 Surveillance Based on Genome k-mers
Source: Microorganisms. 2023 Nov 15;11(11):2773. doi: 10.3390/microorganisms11112773 (PMC10673111; doi:10.3390/microorganisms11112773)
Supplement: Supplementary file 1 [file microorganisms-11-02773-s001.zip › microorganisms-2649077-supplementary.pdf]

Supplementary data

Part 1

In the supplementary data of Part 1 (Tables S1–S5), we provided the results of the text method test when only VOC was considered as a critical variant.

Table S1. Evaluation metrics of new variant detection.

Model	MCC	F1_score	Precision	Recall	Accuracy	Specificity	AUC
AutoEncoder [34]	−0.031	0.000	0.000	0.000	0.858	0.863	0.431
	−0.008	0.013	0.007	0.235	0.720	0.724	0.480
	−0.009	0.000	0.000	0.000	0.937	0.938	0.469
ECOD [30]	−0.029	0.000	0.000	0.000	0.868	0.873	0.436
	0.023	0.025	0.013	0.235	0.850	0.855	0.545
	−0.009	0.000	0.000	0.000	0.945	0.946	0.473
IForest [33]	−0.030	0.000	0.000	0.000	0.864	0.869	0.434
	0.002	0.016	0.008	0.235	0.769	0.773	0.504
	−0.009	0.000	0.000	0.000	0.938	0.939	0.470
KNN [32]	0.107	0.048	0.025	0.667	0.842	0.843	0.755
	0.129	0.048	0.024	1.000	0.678	0.676	0.838
	0.042	0.010	0.005	0.556	0.858	0.859	0.707
LUNAR [35]	0.037	0.024	0.013	0.333	0.840	0.843	0.588
	0.183	0.080	0.041	1.000	0.813	0.812	0.906
	0.019	0.006	0.003	0.333	0.857	0.858	0.596
OCSVM [31]	−0.028	0.000	0.000	0.000	0.879	0.885	0.442
	0.007	0.018	0.010	0.176	0.847	0.853	0.515
	0.040	0.011	0.005	0.444	0.896	0.897	0.671

Table S2. Evaluation metrics of critical variant detection.

Model	MCC	F1_score	Precision	Recall	Accuracy	Specificity	AUC
AutoEncoder [34]	0.504	0.719	0.808	0.647	0.747	0.847	0.747
	0.550	0.724	0.869	0.620	0.763	0.907	0.763
	0.503	0.683	0.859	0.567	0.737	0.907	0.737
	0.548	0.726	0.862	0.627	0.763	0.900	0.763
	0.591	0.758	0.877	0.667	0.787	0.907	0.787
ECOD [30]	0.118	0.346	0.621	0.240	0.547	0.853	0.547
	0.112	0.257	0.649	0.160	0.537	0.913	0.537
	0.089	0.254	0.615	0.160	0.530	0.900	0.530
	0.109	0.265	0.641	0.167	0.537	0.907	0.537
	0.156	0.325	0.681	0.213	0.557	0.900	0.557
IForest [33]	0.150	0.371	0.650	0.260	0.560	0.860	0.560
	0.091	0.246	0.622	0.153	0.530	0.907	0.530
	0.240	0.408	0.750	0.280	0.593	0.907	0.593
	0.138	0.315	0.660	0.207	0.550	0.893	0.550
	0.343	0.523	0.806	0.387	0.647	0.907	0.647
KNN [32]	0.129	0.341	0.636	0.233	0.550	0.867	0.550
	0.243	0.402	0.759	0.273	0.593	0.913	0.593
	0.204	0.347	0.739	0.227	0.573	0.920	0.573
	0.113	0.289	0.636	0.187	0.540	0.893	0.540
	0.169	0.348	0.686	0.233	0.563	0.893	0.563
LUNAR [35]	0.596	0.774	0.855	0.707	0.793	0.880	0.793
	0.696	0.829	0.912	0.760	0.843	0.927	0.843
	0.669	0.806	0.915	0.720	0.827	0.933	0.827
	0.702	0.844	0.878	0.813	0.850	0.887	0.850
	0.651	0.801	0.893	0.727	0.820	0.913	0.820
OCSVM [31]	−0.029	0.207	0.465	0.133	0.490	0.847	0.490
	0.033	0.188	0.548	0.113	0.510	0.907	0.510
	0.000	0.167	0.500	0.100	0.500	0.900	0.500
	−0.047	0.125	0.423	0.073	0.487	0.900	0.487
	0.000	0.185	0.500	0.113	0.500	0.887	0.500

Table S3. Evaluation metrics of new critical variant detection.

Model	MCC	F1_score	Precision	Recall	Accuracy	Specificity	AUC
AutoEncoder [34]	−0.025	0.000	0.000	0.000	0.860	0.863	0.432
	−0.008	0.013	0.007	0.235	0.720	0.724	0.480
	−0.009	0.000	0.000	0.000	0.937	0.938	0.469
ECOD [30]	−0.024	0.000	0.000	0.000	0.870	0.873	0.437
	0.023	0.025	0.013	0.235	0.850	0.855	0.545
	−0.009	0.000	0.000	0.000	0.945	0.946	0.473
IForest [33]	−0.024	0.000	0.000	0.000	0.870	0.873	0.437
	0.008	0.018	0.009	0.294	0.742	0.746	0.520
	−0.009	0.000	0.000	0.000	0.939	0.940	0.470
KNN [32]	0.144	0.048	0.025	1.000	0.844	0.843	0.922
	0.129	0.048	0.024	1.000	0.678	0.676	0.838
	0.042	0.010	0.005	0.556	0.858	0.859	0.707
LUNAR [35]	0.051	0.021	0.011	0.500	0.814	0.815	0.658
	0.227	0.112	0.059	1.000	0.872	0.871	0.935
	0.034	0.007	0.003	0.667	0.748	0.748	0.707
OCSVM [31]	−0.023	0.000	0.000	0.000	0.881	0.885	0.442
	0.007	0.018	0.010	0.176	0.847	0.853	0.515
	0.040	0.011	0.005	0.444	0.896	0.897	0.671
AutoEncoder+AutoEncoder	−0.025	0.000	0.000	0.000	0.860	0.863	0.432
	−0.008	0.013	0.007	0.235	0.720	0.724	0.480
	0.000	0.000	0.000	0.000	0.999	1.000	0.500
AutoEncoder+ECOD	−0.024	0.000	0.000	0.000	0.872	0.875	0.438
	−0.021	0.004	0.002	0.029	0.891	0.898	0.464
	−0.001	0.000	0.000	0.000	0.997	0.999	0.499
AutoEncoder+IForest	−0.024	0.000	0.000	0.000	0.868	0.871	0.436
	0.010	0.019	0.010	0.235	0.802	0.807	0.521
	0.000	0.000	0.000	0.000	0.999	1.000	0.500
AutoEncoder+KNN	−0.024	0.000	0.000	0.000	0.872	0.875	0.438
	−0.008	0.013	0.007	0.235	0.722	0.726	0.481
	0.000	0.000	0.000	0.000	0.999	1.000	0.500
AutoEncoder+LUNAR	−0.025	0.000	0.000	0.000	0.864	0.867	0.434
	−0.008	0.013	0.007	0.235	0.720	0.724	0.480
	0.000	0.000	0.000	0.000	0.999	1.000	0.500
AutoEncoder+OCSVM	−0.023	0.000	0.000	0.000	0.881	0.885	0.442
	0.014	0.021	0.011	0.206	0.844	0.849	0.527
	−0.009	0.000	0.000	0.000	0.937	0.938	0.469
ECOD+AutoEncoder	−0.024	0.000	0.000	0.000	0.872	0.875	0.438
	0.023	0.025	0.013	0.235	0.850	0.855	0.545
	0.000	0.000	0.000	0.000	0.999	1.000	0.500
ECOD+ECOD	−0.024	0.000	0.000	0.000	0.872	0.875	0.438
	−0.013	0.008	0.005	0.059	0.889	0.895	0.477

	−0.001	0.000	0.000	0.000	0.997	0.999	0.499
ECOD+IForest	−0.024	0.000	0.000	0.000	0.870	0.873	0.437
	0.023	0.025	0.013	0.235	0.850	0.855	0.545
	0.000	0.000	0.000	0.000	0.999	1.000	0.500
ECOD+KNN	−0.023	0.000	0.000	0.000	0.875	0.879	0.439
	0.023	0.025	0.013	0.235	0.850	0.855	0.545
	0.000	0.000	0.000	0.000	0.999	1.000	0.500
ECOD+LUNAR	−0.024	0.000	0.000	0.000	0.872	0.875	0.438
	0.023	0.025	0.013	0.235	0.850	0.855	0.545
	0.000	0.000	0.000	0.000	0.999	1.000	0.500
ECOD+OCSVM	−0.023	0.000	0.000	0.000	0.881	0.885	0.442
	0.017	0.022	0.012	0.206	0.855	0.860	0.533
	−0.009	0.000	0.000	0.000	0.945	0.946	0.473
IForest+AutoEncoder	−0.023	0.000	0.000	0.000	0.877	0.881	0.440
	0.017	0.022	0.011	0.235	0.830	0.835	0.535
	0.000	0.000	0.000	0.000	0.999	1.000	0.500
IForest+ECOD	−0.023	0.000	0.000	0.000	0.875	0.879	0.439
	−0.022	0.004	0.002	0.029	0.891	0.898	0.464
	−0.001	0.000	0.000	0.000	0.997	0.999	0.499
IForest+IForest	−0.023	0.000	0.000	0.000	0.877	0.881	0.440
	0.013	0.020	0.011	0.206	0.840	0.845	0.526
	0.000	0.000	0.000	0.000	0.999	1.000	0.500
IForest+KNN	−0.024	0.000	0.000	0.000	0.874	0.877	0.438
	0.013	0.020	0.010	0.294	0.762	0.765	0.530
	0.000	0.000	0.000	0.000	0.999	1.000	0.500
IForest+LUNAR	−0.023	0.000	0.000	0.000	0.875	0.879	0.439
	0.022	0.024	0.012	0.265	0.825	0.830	0.547
	0.000	0.000	0.000	0.000	0.999	1.000	0.500
IForest+OCSVM	−0.023	0.000	0.000	0.000	0.881	0.885	0.442
	0.013	0.021	0.011	0.206	0.843	0.848	0.527
	−0.009	0.000	0.000	0.000	0.939	0.940	0.470
KNN+AutoEncoder	−0.024	0.000	0.000	0.000	0.870	0.873	0.437
	0.018	0.021	0.011	0.324	0.759	0.763	0.543
	0.000	0.000	0.000	0.000	0.999	1.000	0.500
KNN+ECOD	−0.023	0.000	0.000	0.000	0.875	0.879	0.439
	−0.021	0.004	0.002	0.029	0.894	0.901	0.465
	−0.003	0.000	0.000	0.000	0.991	0.993	0.496
KNN+IForest	−0.023	0.000	0.000	0.000	0.875	0.879	0.439
	0.006	0.018	0.009	0.235	0.789	0.794	0.514
	0.000	0.000	0.000	0.000	0.999	1.000	0.500
KNN+KNN	0.144	0.048	0.025	1.000	0.844	0.843	0.922
	0.099	0.045	0.023	0.735	0.749	0.749	0.742
	0.000	0.000	0.000	0.000	0.999	1.000	0.500

KNN+LUNAR	0.065	0.027	0.014	0.500	0.858	0.859	0.680
	0.129	0.048	0.024	1.000	0.679	0.676	0.838
	0.000	0.000	0.000	0.000	0.999	1.000	0.500
KNN+OCSVM	−0.022	0.000	0.000	0.000	0.885	0.889	0.444
	0.012	0.020	0.011	0.206	0.839	0.844	0.525
	0.042	0.010	0.005	0.556	0.858	0.859	0.707
LUNAR+AutoEncoder	−0.025	0.000	0.000	0.000	0.860	0.863	0.432
	0.050	0.037	0.019	0.324	0.862	0.867	0.595
	0.000	0.000	0.000	0.000	0.999	1.000	0.500
LUNAR+ECOD	−0.024	0.000	0.000	0.000	0.874	0.877	0.438
	−0.002	0.014	0.007	0.088	0.898	0.905	0.496
	−0.005	0.000	0.000	0.000	0.981	0.983	0.491
LUNAR+IForest	−0.024	0.000	0.000	0.000	0.874	0.877	0.438
	0.010	0.019	0.010	0.206	0.831	0.836	0.521
	0.000	0.000	0.000	0.000	0.999	1.000	0.500
LUNAR+KNN	0.063	0.026	0.014	0.500	0.854	0.855	0.678
	0.162	0.086	0.046	0.735	0.874	0.875	0.805
	0.000	0.000	0.000	0.000	0.999	1.000	0.500
LUNAR+LUNAR	0.125	0.038	0.019	1.000	0.800	0.800	0.900
	0.206	0.103	0.055	0.912	0.872	0.872	0.892
	0.000	0.000	0.000	0.000	0.999	1.000	0.500
LUNAR+OCSVM	−0.022	0.000	0.000	0.000	0.883	0.887	0.443
	0.024	0.026	0.014	0.206	0.875	0.881	0.543
	0.033	0.007	0.003	0.667	0.744	0.744	0.705
OCSVM+AutoEncoder	−0.023	0.000	0.000	0.000	0.881	0.885	0.442
	0.007	0.018	0.010	0.176	0.848	0.853	0.515
	0.000	0.000	0.000	0.000	0.999	1.000	0.500
OCSVM+ECOD	−0.023	0.000	0.000	0.000	0.881	0.885	0.442
	−0.014	0.008	0.005	0.059	0.888	0.895	0.477
	−0.002	0.000	0.000	0.000	0.994	0.995	0.498
OCSVM+IForest	−0.022	0.000	0.000	0.000	0.883	0.887	0.443
	0.008	0.018	0.010	0.176	0.848	0.853	0.515
	0.000	0.000	0.000	0.000	0.999	1.000	0.500
OCSVM+KNN	−0.022	0.000	0.000	0.000	0.885	0.889	0.444
	0.007	0.018	0.010	0.176	0.847	0.853	0.515
	0.000	0.000	0.000	0.000	0.999	1.000	0.500
OCSVM+LUNAR	−0.022	0.000	0.000	0.000	0.883	0.887	0.443
	0.007	0.018	0.010	0.176	0.847	0.853	0.515
	0.000	0.000	0.000	0.000	0.999	1.000	0.500
OCSVM+OCSVM	−0.023	0.000	0.000	0.000	0.881	0.885	0.442
	0.007	0.018	0.010	0.176	0.847	0.853	0.515
	0.040	0.011	0.005	0.444	0.896	0.897	0.671

Table S4. Evaluation metrics of the detection of all critical variants on the days they first appeared in three countries.

country		China														
clade		21J	21K	22C	20J	20I	23A	20H	22D	22F	22B	22E	21A	21L	22A	21I
date		2021/4/22	2021/12/8	2022/5/30	2021/4/17	2021/1/17	2022/12/9	2020/12/31	2022/1/31	2022/9/27	2022/5/6	2022/9/5	2020/8/10	2021/12/27	2022/6/24	2020/8/10
Number of critical variants on the day they first appeared		1	1	1	2	2	1	1	1	1	1	1	3	1	1	2
Number of samples on the day the critical variants first appeared		1	7	9	2	2	158	1	5	3	3	5	5	1	1	5
Number of samples during 30 days before the day the critical variants first appeared		97	255	608	95	33	1895	7	184	139	439	547	6	72	270	6
KNN	MCC	0.000	0.471	0.000	0.000	0.000	−0.038	0.000	0.408	0.000	0.000	1.000	0.000	0.000	0.000	0.000
	F1_score	1.000	0.500	0.000	1.000	1.000	0.000	0.000	0.500	0.800	0.000	1.000	1.000	1.000	1.000	1.000
	Precision	1.000	0.333	0.000	1.000	1.000	0.000	0.000	0.333	0.667	0.000	1.000	1.000	1.000	1.000	1.000
	Recall	1.000	1.000	0.000	1.000	1.000	0.000	0.000	1.000	1.000	0.000	1.000	1.000	1.000	1.000	1.000
	Accuracy	1.000	0.714	0.889	1.000	1.000	0.810	0.000	0.600	0.667	0.667	1.000	1.000	1.000	1.000	1.000
	Specificity	0.000	0.667	1.000	0.000	0.000	0.815	0.000	0.500	0.000	1.000	1.000	0.000	0.000	0.000	0.000
	AUC	/	0.833	0.500	/	/	0.408	/	0.750	0.500	0.500	1.000	/	/	/	/
LUNAR	MCC	0.000	0.645	1.000	0.000	0.000	−0.026	0.000	1.000	0.000	0.000	0.612	0.000	0.000	0.000	0.000
	F1_score	1.000	0.667	1.000	1.000	1.000	0.000	1.000	1.000	0.800	0.000	0.667	1.000	1.000	1.000	1.000
	Precision	1.000	0.500	1.000	1.000	1.000	0.000	1.000	1.000	0.667	0.000	0.500	1.000	1.000	1.000	1.000
	Recall	1.000	1.000	1.000	1.000	1.000	0.000	1.000	1.000	1.000	0.000	1.000	1.000	1.000	1.000	1.000
	Accuracy	1.000	0.857	1.000	1.000	1.000	0.899	1.000	1.000	0.667	0.667	0.800	1.000	1.000	1.000	1.000
	Specificity	0.000	0.833	1.000	0.000	0.000	0.904	0.000	1.000	0.000	1.000	0.750	0.000	0.000	0.000	0.000
	AUC	/	0.917	1.000	/	/	0.452	/	1.000	0.500	0.500	0.875	/	/	/	/
KNN+LUNAR	MCC	0.000	0.471	0.000	0.000	0.000	−0.038	0.000	0.408	0.000	0.000	1.000	0.000	0.000	0.000	0.000
	F1_score	1.000	0.500	0.000	1.000	0.667	0.000	0.000	0.500	0.800	0.000	1.000	1.000	1.000	1.000	1.000
	Precision	1.000	0.333	0.000	1.000	1.000	0.000	0.000	0.333	0.667	0.000	1.000	1.000	1.000	1.000	1.000
	Recall	1.000	1.000	0.000	1.000	0.500	0.000	0.000	1.000	1.000	0.000	1.000	1.000	1.000	1.000	1.000
	Accuracy	1.000	0.714	0.889	1.000	0.500	0.810	0.000	0.600	0.667	0.667	1.000	1.000	1.000	1.000	1.000
	Specificity	0.000	0.667	1.000	0.000	0.000	0.815	0.000	0.500	0.000	1.000	1.000	0.000	0.000	0.000	0.000
	AUC	/	0.833	0.500	/	/	0.408	/	0.750	0.500	0.500	1.000	/	/	/	/
KNN+KNN	MCC	0.000	0.645	0.000	0.000	0.000	−0.038	0.000	0.408	0.000	0.000	1.000	0.000	0.000	0.000	0.000

	F1_score	0.000	0.667	0.000	1.000	0.667	0.000	0.000	0.500	0.800	0.000	1.000	0.571	1.000	1.000	0.571		
	Precision	0.000	0.500	0.000	1.000	1.000	0.000	0.000	0.333	0.667	0.000	1.000	1.000	1.000	1.000	1.000		
	Recall	0.000	1.000	0.000	1.000	0.500	0.000	0.000	1.000	1.000	0.000	1.000	0.400	1.000	1.000	0.400		
	Accuracy	0.000	0.857	0.889	1.000	0.500	0.810	0.000	0.600	0.667	0.667	1.000	0.400	1.000	1.000	0.400		
	Specificity	0.000	0.833	1.000	0.000	0.000	0.815	0.000	0.500	0.000	1.000	1.000	0.000	0.000	0.000	0.000		
	AUC	/	0.917	0.500	/	/	0.408	/	0.750	0.500	0.500	1.000	/	/	/	/		
	MCC	0.000	0.645	0.000	0.000	0.000	−0.023	0.000	1.000	0.000	0.500	0.612	0.000	0.000	0.000	0.000		
	F1_score	1.000	0.667	0.000	1.000	0.667	0.000	1.000	1.000	0.800	0.667	0.667	1.000	1.000	1.000	1.000		
	Precision	1.000	0.500	0.000	1.000	1.000	0.000	1.000	1.000	0.667	0.500	0.500	1.000	1.000	1.000	1.000		
LUNAR+LUNAR	Recall	1.000	1.000	0.000	1.000	0.500	0.000	1.000	1.000	1.000	1.000	1.000	1.000	1.000	1.000	1.000		
	Accuracy	1.000	0.857	0.889	1.000	0.500	0.918	1.000	1.000	0.667	0.667	0.800	1.000	1.000	1.000	1.000		
	Specificity	0.000	0.833	1.000	0.000	0.000	0.924	0.000	1.000	0.000	0.500	0.750	0.000	0.000	0.000	0.000		
	AUC	/	0.917	0.500	/	/	0.462	/	1.000	0.500	0.750	0.875	/	/	/	/		
	MCC	0.000	0.471	0.000	0.000	0.000	−0.038	0.000	0.408	0.000	0.000	1.000	0.000	0.000	0.000	0.000		
	F1_score	1.000	0.500	0.000	1.000	1.000	0.000	0.000	0.500	0.800	0.000	1.000	1.000	1.000	1.000	1.000		
	Precision	1.000	0.333	0.000	1.000	1.000	0.000	0.000	0.333	0.667	0.000	1.000	1.000	1.000	1.000	1.000		
step1KNN	Recall	1.000	1.000	0.000	1.000	1.000	0.000	0.000	1.000	1.000	0.000	1.000	1.000	1.000	1.000	1.000		
	Accuracy	1.000	0.714	0.889	1.000	1.000	0.810	0.000	0.600	0.667	0.667	1.000	1.000	1.000	1.000	1.000		
	Specificity	0.000	0.667	1.000	0.000	0.000	0.815	0.000	0.500	0.000	1.000	1.000	0.000	0.000	0.000	0.000		
	AUC	/	0.833	0.500	/	/	0.408	/	0.750	0.500	0.500	1.000	/	/	/	/		
	MCC	0.000	0.645	1.000	0.000	0.000	0.168	0.000	1.000	0.000	0.000	0.612	0.000	0.000	0.000	0.000		
	F1_score	1.000	0.667	1.000	1.000	1.000	0.067	1.000	1.000	0.800	0.500	0.667	1.000	1.000	1.000	1.000		
	Precision	1.000	0.500	1.000	1.000	1.000	0.034	1.000	1.000	0.667	0.333	0.500	1.000	1.000	1.000	1.000		
step1LUNAR	Recall	1.000	1.000	1.000	1.000	1.000	1.000	1.000	1.000	1.000	1.000	1.000	1.000	1.000	1.000	1.000		
	Accuracy	1.000	0.857	1.000	1.000	1.000	0.823	1.000	1.000	0.667	0.333	0.800	1.000	1.000	1.000	1.000		
	Specificity	0.000	0.833	1.000	0.000	0.000	0.822	0.000	1.000	0.000	0.000	0.750	0.000	0.000	0.000	0.000		
	AUC	/	0.917	1.000	/	/	0.911	/	1.000	0.500	0.500	0.875	/	/	/	/		

country	Portugal																
clade	22F	22C	22B	20I	20J	21L	22E	21I	21J	21A	22D	21K	23C	22A	23A	21M	20H
date	2022/10/8	2022/4/25	2022/3/29	2020/11/9	2021/2/9	2022/1/2	2022/9/7	2021/5/6	2021/4/21	2021/4/18	2022/8/14	2021/2/2	2022/10/21	2022/5/16	2022/12/5	2022/3/7	2020/12/26
Number of critical variants on the day they first appeared	1	1	1	1	1	1	1	1	1	1	1	1	1	1	1	1	1
Number of samples on the day the critical variants first	40	174	156	55	164	146	6	106	9	21	26	8	54	181	60	214	72

appeared																		
Number of samples during 30 days before the day the critical variants first appeared		1195	2333	2533	29	1189	1950	1133	1850	1691	1641	1121	1118	833	2409	758	2118	200
KNN	MCC	0.381	0.170	0.209	−0.019	0.176	−0.007	−0.200	−0.030	0.661	0.000	−0.040	0.655	−0.027	0.191	−0.039	0.269	−0.025
	F1_score	0.286	0.067	0.095	0.000	0.071	0.000	0.000	0.000	0.667	0.000	0.000	0.667	0.000	0.080	0.000	0.143	0.000
	Precision	0.167	0.034	0.050	0.000	0.037	0.000	0.000	0.000	0.500	0.000	0.000	0.500	0.000	0.042	0.000	0.077	0.000
	Recall	1.000	1.000	1.000	0.000	1.000	0.000	0.000	0.000	1.000	0.000	0.000	1.000	0.000	1.000	0.000	1.000	0.000
	Accuracy	0.875	0.839	0.878	0.964	0.841	0.986	0.667	0.906	0.889	0.952	0.923	0.875	0.944	0.873	0.900	0.944	0.944
	Specificity	0.872	0.838	0.877	0.981	0.840	0.993	0.800	0.914	0.875	1.000	0.960	0.857	0.962	0.872	0.915	0.944	0.958
	AUC	0.936	0.919	0.939	0.491	0.920	0.497	0.400	0.457	0.938	0.500	0.480	0.929	0.481	0.936	0.458	0.972	0.479
LUNAR	MCC	0.348	0.181	0.216	−0.019	0.169	0.705	−0.200	−0.033	0.661	0.689	1.000	0.655	−0.039	0.098	−0.039	0.259	−0.025
	F1_score	0.250	0.074	0.100	0.000	0.067	0.667	0.000	0.000	0.667	0.667	1.000	0.667	0.000	0.030	0.000	0.133	0.000
	Precision	0.143	0.038	0.053	0.000	0.034	0.500	0.000	0.000	0.500	0.500	1.000	0.500	0.000	0.015	0.000	0.071	0.000
	Recall	1.000	1.000	1.000	0.000	1.000	1.000	0.000	0.000	1.000	1.000	1.000	1.000	0.000	1.000	0.000	1.000	0.000
	Accuracy	0.850	0.856	0.885	0.964	0.829	0.993	0.667	0.887	0.889	0.952	1.000	0.875	0.907	0.641	0.900	0.939	0.944
	Specificity	0.846	0.855	0.884	0.981	0.828	0.993	0.800	0.895	0.875	0.950	1.000	0.857	0.925	0.639	0.915	0.939	0.958
	AUC	0.923	0.928	0.942	0.491	0.914	0.997	0.400	0.448	0.938	0.975	1.000	0.929	0.462	0.819	0.458	0.969	0.479
KNN+LUNAR	MCC	0.381	0.170	0.209	−0.019	0.223	−0.007	−0.200	−0.030	0.661	0.000	−0.040	1.000	−0.027	0.191	−0.039	0.269	−0.025
	F1_score	0.286	0.067	0.095	0.000	0.105	0.000	0.000	0.000	0.667	0.000	0.000	1.000	0.000	0.080	0.000	0.143	0.000
	Precision	0.167	0.034	0.050	0.000	0.056	0.000	0.000	0.000	0.500	0.000	0.000	1.000	0.000	0.042	0.000	0.077	0.000
	Recall	1.000	1.000	1.000	0.000	1.000	0.000	0.000	0.000	1.000	0.000	0.000	1.000	0.000	1.000	0.000	1.000	0.000
	Accuracy	0.875	0.839	0.878	0.964	0.896	0.986	0.667	0.906	0.889	0.952	0.923	1.000	0.944	0.873	0.900	0.944	0.944
	Specificity	0.872	0.838	0.877	0.981	0.896	0.993	0.800	0.914	0.875	1.000	0.960	1.000	0.962	0.872	0.915	0.944	0.958
	AUC	0.936	0.919	0.939	0.491	0.948	0.497	0.400	0.457	0.938	0.500	0.480	1.000	0.481	0.936	0.458	0.972	0.479
KNN+KNN	MCC	0.381	0.174	0.209	−0.019	0.247	−0.007	−0.200	−0.030	0.661	0.000	−0.040	1.000	−0.027	−0.026	−0.039	−0.015	−0.025
	F1_score	0.286	0.069	0.095	0.000	0.125	0.000	0.000	0.000	0.667	0.000	0.000	1.000	0.000	0.000	0.000	0.000	0.000
	Precision	0.167	0.036	0.050	0.000	0.067	0.000	0.000	0.000	0.500	0.000	0.000	1.000	0.000	0.000	0.000	0.000	0.000
	Recall	1.000	1.000	1.000	0.000	1.000	0.000	0.000	0.000	1.000	0.000	0.000	1.000	0.000	0.000	0.000	0.000	0.000
	Accuracy	0.875	0.845	0.878	0.964	0.915	0.986	0.667	0.906	0.889	0.952	0.923	1.000	0.944	0.884	0.900	0.949	0.944
	Specificity	0.872	0.844	0.877	0.981	0.914	0.993	0.800	0.914	0.875	1.000	0.960	1.000	0.962	0.889	0.915	0.953	0.958
	AUC	0.936	0.922	0.939	0.491	0.957	0.497	0.400	0.457	0.938	0.500	0.480	1.000	0.481	0.444	0.458	0.477	0.479
LUNAR+LUNAR	MCC	0.381	0.181	0.209	−0.019	0.210	0.705	−0.200	−0.033	0.661	0.000	1.000	1.000	−0.049	0.239	−0.035	0.259	−0.025
	F1_score	0.286	0.074	0.095	0.000	0.095	0.667	0.000	0.000	0.667	0.000	1.000	1.000	0.000	0.118	0.000	0.133	0.000

	Precision	0.167	0.038	0.050	0.000	0.050	0.500	0.000	0.000	0.500	0.000	1.000	1.000	0.000	0.063	0.000	0.071	0.000
	Recall	1.000	1.000	1.000	0.000	1.000	1.000	0.000	0.000	1.000	0.000	1.000	1.000	0.000	1.000	0.000	1.000	0.000
	Accuracy	0.875	0.856	0.878	0.964	0.884	0.993	0.667	0.887	0.889	0.952	1.000	1.000	0.870	0.917	0.917	0.939	0.944
	Specificity	0.872	0.855	0.877	0.981	0.883	0.993	0.800	0.895	0.875	1.000	1.000	1.000	0.887	0.917	0.932	0.939	0.958
	AUC	0.936	0.928	0.939	0.491	0.942	0.997	0.400	0.448	0.938	0.500	1.000	1.000	0.443	0.958	0.466	0.969	0.479
	MCC	0.381	0.170	0.209	−0.019	0.176	−0.007	−0.200	−0.030	0.661	0.000	−0.040	0.655	−0.027	0.191	−0.039	0.269	−0.025
	F1_score	0.286	0.067	0.095	0.000	0.071	0.000	0.000	0.000	0.667	0.000	0.000	0.667	0.000	0.080	0.000	0.143	0.000
	Precision	0.167	0.034	0.050	0.000	0.037	0.000	0.000	0.000	0.500	0.000	0.000	0.500	0.000	0.042	0.000	0.077	0.000
step1KNN	Recall	1.000	1.000	1.000	0.000	1.000	0.000	0.000	0.000	1.000	0.000	0.000	1.000	0.000	1.000	0.000	1.000	0.000
	Accuracy	0.875	0.839	0.878	0.964	0.841	0.986	0.667	0.906	0.889	0.952	0.923	0.875	0.944	0.873	0.900	0.944	0.944
	Specificity	0.872	0.838	0.877	0.981	0.840	0.993	0.800	0.914	0.875	1.000	0.960	0.857	0.962	0.872	0.915	0.944	0.958
	AUC	0.936	0.919	0.939	0.491	0.920	0.497	0.400	0.457	0.938	0.500	0.480	0.929	0.481	0.936	0.458	0.972	0.479
	MCC	0.381	0.186	0.204	−0.019	0.176	0.705	−0.200	0.155	0.661	0.000	1.000	0.655	−0.039	0.224	−0.039	0.250	−0.025
	F1_score	0.286	0.077	0.091	0.000	0.071	0.667	0.000	0.065	0.667	0.000	1.000	0.667	0.000	0.105	0.000	0.125	0.000
	Precision	0.167	0.040	0.048	0.000	0.037	0.500	0.000	0.033	0.500	0.000	1.000	0.500	0.000	0.056	0.000	0.067	0.000
step1LUNAR	Recall	1.000	1.000	1.000	0.000	1.000	1.000	0.000	1.000	1.000	0.000	1.000	1.000	0.000	1.000	0.000	1.000	0.000
	Accuracy	0.875	0.862	0.872	0.964	0.841	0.993	0.667	0.726	0.889	0.952	1.000	0.875	0.907	0.906	0.900	0.935	0.944
	Specificity	0.872	0.861	0.871	0.981	0.840	0.993	0.800	0.724	0.875	1.000	1.000	0.857	0.925	0.906	0.915	0.934	0.958
	AUC	0.936	0.931	0.935	0.491	0.920	0.997	0.400	0.862	0.938	0.500	1.000	0.929	0.462	0.953	0.458	0.967	0.479

country		Argentina																
clade		20H	20I	20J	21A	21I	21J	21K	21L	21M	22A	22B	22C	22D	22E	22F	23A	23C
date		2021/4/24	2020/12/21	2020/7/22	2021/4/24	2021/6/4	2020/11/9	2021/1/6	2022/1/18	2021/12/21	2022/5/9	2022/5/21	2022/3/16	2022/1/2	2022/5/22	2022/10/12	2022/12/6	2022/12/5
Number of critical variants on the day they first appeared		1	1	1	1	1	1	1	1	4	1	1	1	1	1	1	1	2
Number of samples on the day the critical variants first appeared		18	15	13	18	31	4	11	54	133	9	7	8	27	5	3	44	65
Number of samples during 30 days before the day the critical variants first appeared		742	142	192	742	802	165	155	1924	1764	155	278	359	2071	279	110	309	246
	MCC	0.000	−0.071	0.527	0.000	−0.033	0.577	0.671	0.307	0.460	−0.189	−0.167	0.655	0.411	0.000	0.000	−0.066	−0.039
	F1_score	0.000	0.000	0.500	0.000	0.000	0.667	0.667	0.200	0.381	0.000	0.000	0.667	0.333	0.000	0.000	0.000	0.000
KNN	Precision	0.000	0.000	0.333	0.000	0.000	0.500	0.500	0.111	0.235	0.000	0.000	0.500	0.200	0.000	0.000	0.000	0.000
	Recall	0.000	0.000	1.000	0.000	0.000	1.000	1.000	1.000	1.000	0.000	0.000	1.000	1.000	0.000	0.000	0.000	0.000
	Accuracy	0.889	0.867	0.846	0.889	0.935	0.750	0.909	0.852	0.902	0.667	0.714	0.875	0.852	0.800	0.667	0.818	0.923

	Specificity	1.000	0.929	0.833	1.000	0.967	0.667	0.900	0.849	0.899	0.750	0.833	0.857	0.846	1.000	1.000	0.837	0.952
	AUC	0.500	0.464	0.917	0.500	0.483	0.833	0.950	0.925	0.950	0.375	0.417	0.929	0.923	0.500	0.500	0.419	0.476
	MCC	0.000	−0.105	0.527	0.000	0.558	0.577	1.000	0.389	0.654	−0.189	0.000	0.000	0.555	0.000	0.000	−0.072	−0.039
	F1_score	0.000	0.000	0.500	0.000	0.500	0.667	1.000	0.286	0.615	0.000	0.000	0.000	0.500	0.000	0.000	0.000	0.000
	Precision	0.000	0.000	0.333	0.000	0.333	0.500	1.000	0.167	0.444	0.000	0.000	0.000	0.333	0.000	0.000	0.000	0.000
LUNAR	Recall	0.000	0.000	1.000	0.000	1.000	1.000	1.000	1.000	1.000	0.000	0.000	0.000	1.000	0.000	0.000	0.000	0.000
	Accuracy	0.889	0.800	0.846	0.889	0.935	0.750	1.000	0.907	0.962	0.667	0.857	0.875	0.926	0.800	0.667	0.795	0.923
	Specificity	1.000	0.857	0.833	1.000	0.933	0.667	1.000	0.906	0.961	0.750	1.000	1.000	0.923	1.000	1.000	0.814	0.952
	AUC	0.500	0.429	0.917	0.500	0.967	0.833	1.000	0.953	0.981	0.375	0.500	0.500	0.962	0.500	0.500	0.407	0.476
	MCC	0.000	−0.071	0.527	0.000	−0.033	0.577	0.671	0.307	0.476	−0.189	−0.167	0.655	0.411	0.000	0.000	−0.066	−0.039
	F1_score	0.000	0.000	0.500	0.000	0.000	0.667	0.667	0.200	0.400	0.000	0.000	0.667	0.333	0.000	0.000	0.000	0.000
	Precision	0.000	0.000	0.333	0.000	0.000	0.500	0.500	0.111	0.250	0.000	0.000	0.500	0.200	0.000	0.000	0.000	0.000
KNN+LUNAR	Recall	0.000	0.000	1.000	0.000	0.000	1.000	1.000	1.000	1.000	0.000	0.000	1.000	1.000	0.000	0.000	0.000	0.000
	Accuracy	0.889	0.867	0.846	0.889	0.935	0.750	0.909	0.852	0.910	0.667	0.714	0.875	0.852	0.800	0.667	0.818	0.923
	Specificity	1.000	0.929	0.833	1.000	0.967	0.667	0.900	0.849	0.907	0.750	0.833	0.857	0.846	1.000	1.000	0.837	0.952
	AUC	0.500	0.464	0.917	0.500	0.483	0.833	0.950	0.925	0.953	0.375	0.417	0.929	0.923	0.500	0.500	0.419	0.476
	MCC	0.000	−0.071	0.527	0.000	−0.033	0.577	0.671	−0.057	0.460	−0.189	−0.167	1.000	0.411	0.000	0.000	−0.066	−0.039
	F1_score	0.000	0.000	0.500	0.000	0.000	0.667	0.667	0.000	0.381	0.000	0.000	1.000	0.333	0.000	0.000	0.000	0.000
	Precision	0.000	0.000	0.333	0.000	0.000	0.500	0.500	0.000	0.235	0.000	0.000	1.000	0.200	0.000	0.000	0.000	0.000
KNN+KNN	Recall	0.000	0.000	1.000	0.000	0.000	1.000	1.000	0.000	1.000	0.000	0.000	1.000	1.000	0.000	0.000	0.000	0.000
	Accuracy	0.889	0.867	0.846	0.889	0.935	0.750	0.909	0.833	0.902	0.667	0.714	1.000	0.852	0.800	0.667	0.818	0.923
	Specificity	1.000	0.929	0.833	1.000	0.967	0.667	0.900	0.849	0.899	0.750	0.833	1.000	0.846	1.000	1.000	0.837	0.952
	AUC	0.500	0.464	0.917	0.500	0.483	0.833	0.950	0.425	0.950	0.375	0.417	1.000	0.923	0.500	0.500	0.419	0.476
	MCC	0.000	−0.105	0.527	0.000	0.695	0.577	1.000	0.430	0.696	−0.189	0.000	0.000	0.555	0.000	1.000	−0.083	−0.039
	F1_score	0.000	0.000	0.500	0.000	0.667	0.667	1.000	0.333	0.667	0.000	0.000	0.000	0.500	0.000	1.000	0.000	0.000
	Precision	0.000	0.000	0.333	0.000	0.500	0.500	1.000	0.200	0.500	0.000	0.000	0.000	0.333	0.000	1.000	0.000	0.000
LUNAR+LUNAR	Recall	0.000	0.000	1.000	0.000	1.000	1.000	1.000	1.000	1.000	0.000	0.000	0.000	1.000	0.000	1.000	0.000	0.000
	Accuracy	0.889	0.800	0.846	0.889	0.968	0.750	1.000	0.926	0.970	0.667	0.857	0.875	0.926	0.800	1.000	0.750	0.923
	Specificity	1.000	0.857	0.833	1.000	0.967	0.667	1.000	0.925	0.969	0.750	1.000	1.000	0.923	1.000	1.000	0.767	0.952
	AUC	0.500	0.429	0.917	0.500	0.983	0.833	1.000	0.962	0.984	0.375	0.500	0.500	0.962	0.500	1.000	0.384	0.476
	MCC	0.000	−0.071	0.527	0.000	−0.033	0.577	0.671	0.307	0.460	−0.189	−0.167	0.655	0.411	0.000	0.000	−0.066	−0.039
step1KNN	F1_score	0.000	0.000	0.500	0.000	0.000	0.667	0.667	0.200	0.381	0.000	0.000	0.667	0.333	0.000	0.000	0.000	0.000
	Precision	0.000	0.000	0.333	0.000	0.000	0.500	0.500	0.111	0.235	0.000	0.000	0.500	0.200	0.000	0.000	0.000	0.000

	Recall	0.000	0.000	1.000	0.000	0.000	1.000	1.000	1.000	1.000	0.000	0.000	1.000	1.000	0.000	0.000	0.000	0.000
	Accuracy	0.889	0.867	0.846	0.889	0.935	0.750	0.909	0.852	0.902	0.667	0.714	0.875	0.852	0.800	0.667	0.818	0.923
	Specificity	1.000	0.929	0.833	1.000	0.967	0.667	0.900	0.849	0.899	0.750	0.833	0.857	0.846	1.000	1.000	0.837	0.952
	AUC	0.500	0.464	0.917	0.500	0.483	0.833	0.950	0.925	0.950	0.375	0.417	0.929	0.923	0.500	0.500	0.419	0.476
	MCC	0.686	−0.105	0.527	0.000	−0.033	0.577	0.671	0.389	0.654	−0.189	−0.167	0.655	0.555	0.000	0.000	−0.072	−0.046
	F1_score	0.667	0.000	0.500	0.000	0.000	0.667	0.667	0.286	0.615	0.000	0.000	0.667	0.500	0.000	0.000	0.000	0.000
	Precision	1.000	0.000	0.333	0.000	0.000	0.500	0.500	0.167	0.444	0.000	0.000	0.500	0.333	0.000	0.000	0.000	0.000
step1LUNAR	Recall	0.500	0.000	1.000	0.000	0.000	1.000	1.000	1.000	1.000	0.000	0.000	1.000	1.000	0.000	0.000	0.000	0.000
	Accuracy	0.944	0.800	0.846	0.889	0.935	0.750	0.909	0.907	0.962	0.667	0.714	0.875	0.926	0.800	0.667	0.795	0.908
	Specificity	1.000	0.857	0.833	1.000	0.967	0.667	0.900	0.906	0.961	0.750	0.833	0.857	0.923	1.000	1.000	0.814	0.937
	AUC	0.750	0.429	0.917	0.500	0.483	0.833	0.950	0.953	0.981	0.375	0.417	0.929	0.962	0.500	0.500	0.407	0.468

Table S5. Results of analog dynamic monitoring of new critical variant detection.

country	date	Number of samples on the day	Number of samples during 30 days before the day	Real number of the new critical variants	KNN			LUNAR			KNN+LUNAR			KNN+KNN			LUNAR+LUNAR		
					Predicted number of the new critical variants	Recall	Specificity	Predicted number of the new critical variants	Recall	Specificity	Predicted number of the new critical variants	Recall	Specificity	Predicted number of the new critical variants	Recall	Specificity	Predicted number of the new critical variants	Recall	Specificity
Argentina	2021/10/1	18	708	0	5	/	0.722	7	/	0.611	5	/	0.722	5	/	0.722	6	/	0.667
	2021/10/2	16	675	0	3	/	0.813	2	/	0.875	4	/	0.750	4	/	0.750	2	/	0.875
	2021/10/3	5	654	0	2	/	0.600	1	/	0.800	2	/	0.600	2	/	0.600	1	/	0.800
	2021/10/4	23	621	0	5	/	0.783	2	/	0.913	4	/	0.826	4	/	0.826	2	/	0.913
	2021/10/5	19	627	0	0	/	1.000	1	/	0.947	1	/	0.947	1	/	0.947	4	/	0.789
	2021/10/6	25	621	0	3	/	0.880	2	/	0.920	3	/	0.880	3	/	0.880	2	/	0.920
	2021/10/7	31	600	0	1	/	0.968	2	/	0.935	2	/	0.935	2	/	0.935	1	/	0.968
	2021/10/8	22	593	1	3	0	0.857	1	0	0.952	2	0	0.905	2	0	0.905	1	0	0.952
	2021/10/9	13	595	0	0	/	1.000	0	/	1.000	1	/	0.923	1	/	0.923	0	/	1.000
	2021/10/10	20	589	0	1	/	0.950	0	/	1.000	1	/	0.950	1	/	0.950	2	/	0.900
	2021/10/11	18	590	0	1	/	0.944	1	/	0.944	1	/	0.944	1	/	0.944	1	/	0.944
	2021/10/12	55	595	0	2	/	0.964	2	/	0.964	2	/	0.964	2	/	0.964	2	/	0.964
	2021/10/13	35	638	0	2	/	0.943	1	/	0.971	1	/	0.971	1	/	0.971	1	/	0.971
	2021/10/14	51	648	0	4	/	0.922	4	/	0.922	4	/	0.922	4	/	0.922	9	/	0.824
	2021/10/15	47	674	0	4	/	0.915	8	/	0.830	4	/	0.915	4	/	0.915	3	/	0.936
	2021/10/16	31	695	0	1	/	0.968	1	/	0.968	1	/	0.968	1	/	0.968	1	/	0.968

	2021/10/17	12	694	0	0	/	1.000	0	/	1.000	0	/	1.000	0	/	1.000	0	/	1.000
	2021/10/18	60	683	0	2	/	0.967	2	/	0.967	1	/	0.983	1	/	0.983	2	/	0.967
	2021/10/19	52	730	0	4	/	0.923	3	/	0.942	3	/	0.942	3	/	0.942	3	/	0.942
	2021/10/20	53	773	0	4	/	0.925	3	/	0.943	2	/	0.962	3	/	0.943	2	/	0.962
	2021/10/21	57	804	0	6	/	0.895	2	/	0.965	6	/	0.895	6	/	0.895	2	/	0.965
	2021/10/22	58	836	0	10	/	0.828	4	/	0.931	4	/	0.931	4	/	0.931	5	/	0.914
	2021/10/23	34	883	0	3	/	0.912	3	/	0.912	3	/	0.912	3	/	0.912	3	/	0.912
	2021/10/24	24	881	0	2	/	0.917	1	/	0.958	3	/	0.875	3	/	0.875	1	/	0.958
	2021/10/25	68	885	0	7	/	0.897	5	/	0.926	6	/	0.912	6	/	0.912	7	/	0.897
	2021/10/26	80	936	0	6	/	0.925	4	/	0.950	6	/	0.925	6	/	0.925	4	/	0.950
	2021/10/27	72	1005	1	3	1	0.972	3	1	0.972	5	1	0.944	4	1	0.958	3	1	0.972
	2021/10/28	77	1057	0	9	/	0.883	4	/	0.948	7	/	0.909	7	/	0.909	5	/	0.935
	2021/10/29	73	1115	0	10	/	0.863	8	/	0.890	15	/	0.795	11	/	0.849	8	/	0.890
	2021/10/30	30	1169	0	2	/	0.933	3	/	0.900	3	/	0.900	3	/	0.900	1	/	0.967
	2021/10/31	18	1179	0	0	/	1.000	0	/	1.000	0	/	1.000	0	/	1.000	0	/	1.000
	2022/5/1	20	338	0	3	/	0.850	2	/	0.900	3	/	0.850	3	/	0.850	1	/	0.950
China	2022/5/2	21	358	0	4	/	0.810	2	/	0.905	6	/	0.714	6	/	0.714	4	/	0.810
	2022/5/3	27	378	0	2	/	0.926	2	/	0.926	4	/	0.852	4	/	0.852	3	/	0.889

	2022/5/4	26	401	0	2	/	0.923	2	/	0.923	2	/	0.923	2	/	0.923	4	/	0.846
	2022/5/5	28	415	0	7	/	0.750	7	/	0.750	8	/	0.714	8	/	0.714	7	/	0.750
	2022/5/6	3	439	1	0	0	1.000	0	0	1.000	0	0	1.000	0	0	1.000	0	0	1.000
	2022/5/7	20	440	0	4	/	0.800	4	/	0.800	3	/	0.850	3	/	0.850	4	/	0.800
	2022/5/8	28	448	0	0	/	1.000	1	/	0.964	1	/	0.964	1	/	0.964	1	/	0.964
	2022/5/9	13	470	0	2	/	0.846	2	/	0.846	2	/	0.846	2	/	0.846	2	/	0.846
	2022/5/10	14	477	0	3	/	0.786	3	/	0.786	3	/	0.786	3	/	0.786	3	/	0.786
	2022/5/11	15	482	0	1	/	0.933	2	/	0.867	2	/	0.867	2	/	0.867	1	/	0.933
	2022/5/12	31	489	0	2	/	0.935	2	/	0.935	2	/	0.935	2	/	0.935	2	/	0.935
	2022/5/13	16	510	0	1	/	0.938	1	/	0.938	1	/	0.938	1	/	0.938	6	/	0.625
	2022/5/14	19	523	0	1	/	0.947	1	/	0.947	1	/	0.947	1	/	0.947	1	/	0.947
	2022/5/15	23	538	0	0	/	1.000	0	/	1.000	0	/	1.000	0	/	1.000	0	/	1.000
	2022/5/16	26	559	0	0	/	1.000	0	/	1.000	0	/	1.000	0	/	1.000	0	/	1.000
	2022/5/17	14	580	0	0	/	1.000	0	/	1.000	0	/	1.000	0	/	1.000	0	/	1.000
	2022/5/18	24	591	0	0	/	1.000	0	/	1.000	0	/	1.000	0	/	1.000	0	/	1.000
	2022/5/19	21	607	0	0	/	1.000	0	/	1.000	0	/	1.000	0	/	1.000	0	/	1.000
	2022/5/20	31	622	0	0	/	1.000	0	/	1.000	0	/	1.000	0	/	1.000	0	/	1.000
	2022/5/21	25	651	0	0	/	1.000	0	/	1.000	0	/	1.000	0	/	1.000	0	/	1.000
	2022/5/22	32	676	0	0	/	1.000	0	/	1.000	0	/	1.000	0	/	1.000	0	/	1.000
	2022/5/23	18	699	0	0	/	1.000	0	/	1.000	0	/	1.000	0	/	1.000	0	/	1.000
	2022/5/24	17	691	0	0	/	1.000	0	/	1.000	0	/	1.000	0	/	1.000	0	/	1.000
	2022/5/25	20	691	0	0	/	1.000	0	/	1.000	0	/	1.000	0	/	1.000	1	/	0.950
	2022/5/26	17	693	0	0	/	1.000	0	/	1.000	0	/	1.000	0	/	1.000	0	/	1.000
	2022/5/27	12	672	0	0	/	1.000	0	/	1.000	0	/	1.000	0	/	1.000	0	/	1.000
	2022/5/28	11	658	0	0	/	1.000	0	/	1.000	0	/	1.000	0	/	1.000	4	/	0.636
	2022/5/29	7	638	0	0	/	1.000	0	/	1.000	0	/	1.000	0	/	1.000	0	/	1.000
	2022/5/30	9	608	1	0	0	1.000	1	1	1.000	0	0	1.000	0	0	1.000	0	0	1.000
	2022/5/31	19	588	0	0	/	1.000	2	/	0.895	0	/	1.000	0	/	1.000	1	/	0.947
Portugal	2021/4/1	12	1010	0	5	/	0.583	4	/	0.667	5	/	0.583	5	/	0.583	5	/	0.583
	2021/4/2	8	911	0	1	/	0.875	5	/	0.375	1	/	0.875	1	/	0.875	1	/	0.875
	2021/4/3	5	798	0	0	/	1.000	0	/	1.000	0	/	1.000	0	/	1.000	0	/	1.000
	2021/4/4	8	710	0	1	/	0.875	1	/	0.875	1	/	0.875	1	/	0.875	1	/	0.875

	2021/4/5	233	616	0	51	/	0.781	44	/	0.811	51	/	0.781	51	/	0.781	45	/	0.807
	2021/4/6	196	790	0	12	/	0.939	10	/	0.949	13	/	0.934	13	/	0.934	10	/	0.949
	2021/4/7	166	942	0	36	/	0.783	30	/	0.819	38	/	0.771	38	/	0.771	34	/	0.795
	2021/4/8	189	1023	0	8	/	0.958	6	/	0.968	7	/	0.963	7	/	0.963	6	/	0.968
	2021/4/9	200	1154	0	12	/	0.940	8	/	0.960	13	/	0.935	13	/	0.935	10	/	0.950
	2021/4/10	126	1277	0	7	/	0.944	8	/	0.937	10	/	0.921	10	/	0.921	6	/	0.952
	2021/4/11	50	1331	0	1	/	0.980	1	/	0.980	1	/	0.980	1	/	0.980	1	/	0.980
	2021/4/12	78	1335	0	5	/	0.936	6	/	0.923	5	/	0.936	5	/	0.936	6	/	0.923
	2021/4/13	80	1364	0	5	/	0.938	4	/	0.950	5	/	0.938	5	/	0.938	5	/	0.938
	2021/4/14	63	1419	0	6	/	0.905	5	/	0.921	6	/	0.905	6	/	0.905	5	/	0.921
	2021/4/15	82	1469	0	4	/	0.951	3	/	0.963	3	/	0.963	3	/	0.963	3	/	0.963
	2021/4/16	60	1547	0	7	/	0.883	8	/	0.867	8	/	0.867	8	/	0.867	8	/	0.867
	2021/4/17	42	1600	0	0	/	1.000	3	/	0.929	0	/	1.000	0	/	1.000	0	/	1.000
	2021/4/18	21	1641	1	0	0	1.000	0	0	1.000	0	0	1.000	0	0	1.000	0	0	1.000
	2021/4/19	30	1657	0	3	/	0.900	3	/	0.900	3	/	0.900	3	/	0.900	3	/	0.900
	2021/4/20	5	1686	0	1	/	0.800	1	/	0.800	1	/	0.800	1	/	0.800	1	/	0.800
	2021/4/21	9	1691	1	2	1	0.875	2	1	0.875	2	1	0.875	2	1	0.875	2	1	0.875
	2021/4/22	2	1697	0	0	/	1.000	0	/	1.000	0	/	1.000	0	/	1.000	0	/	1.000
	2021/4/23	1	1696	0	0	/	1.000	1	/	0.000	0	/	1.000	0	/	1.000	0	/	1.000
	2021/4/24	6	1696	0	0	/	1.000	0	/	1.000	0	/	1.000	0	/	1.000	0	/	1.000
	2021/4/25	1	1696	0	1	/	0.000	1	/	0.000	1	/	0.000	1	/	0.000	1	/	0.000
	2021/4/26	4	1692	0	2	/	0.500	1	/	0.750	2	/	0.500	2	/	0.500	1	/	0.750
	2021/4/27	11	1695	0	0	/	1.000	1	/	0.909	1	/	0.909	1	/	0.909	0	/	1.000
	2021/4/28	3	1705	0	1	/	0.667	1	/	0.667	1	/	0.667	1	/	0.667	1	/	0.667
	2021/4/29	1	1704	0	1	/	0.000	1	/	0.000	1	/	0.000	1	/	0.000	1	/	0.000
	2021/4/30	8	1697	0	0	/	1.000	0	/	1.000	0	/	1.000	0	/	1.000	0	/	1.000

Part 2

Considering that VOI has now been largely proven to be benign, the text only considers VOC as the critical variant. In the supplementary data below (Tables S6–S9, Figures S1–S4), we provided the results of the text method test when both VOC and VOI were considered as critical variant.

When we considered VOC/VOI as a critical variant, the five better models obtained by testing on our data in the step of new critical variant detection are KNN, LUNAR, KNN+LUNAR, LUNAR+KNN and KNN+KNN. The recall rates of these five models are above 0.5 and the AUC scores are above 0.7. Therefore, we used these five better performing models in our subsequent evaluation. Combined with the rounds of evaluation, LUNAR was the most prominent model of all the tested models. This result is consistent with the results in the text that only consider VOC as a critical variant.

Table S6. Evaluation of new variant detection (consider VOC/VOI as critical variants).

Model	MCC	F1_score	Precision	Recall	Accuracy	Specificity	AUC
AutoEncoder [34]	−0.016	0.004	0.002	0.078	0.838	0.842	0.460
ECOD [30]	−0.005	0.008	0.004	0.078	0.887	0.891	0.485
IForest [33]	−0.004	0.009	0.005	0.088	0.883	0.886	0.487
KNN [32]	0.092	0.035	0.018	0.741	0.793	0.792	0.767
LUNAR [35]	0.117	0.051	0.027	0.852	0.775	0.774	0.813
OCSVM [31]	0.006	0.010	0.005	0.207	0.874	0.878	0.543

Table S7. Evaluation of critical variant detection (consider VOC/VOI as critical variants).

Model	MCC	F1_score	Precision	Recall	Accuracy	Specificity	AUC
AutoEncoder [34]	0.643	0.799	0.881	0.732	0.817	0.901	0.817
ECOD [30]	0.350	0.533	0.806	0.399	0.651	0.903	0.651
IForest [33]	0.332	0.509	0.796	0.380	0.642	0.904	0.642
KNN [32]	0.247	0.424	0.747	0.297	0.599	0.900	0.599
LUNAR [35]	0.655	0.801	0.900	0.721	0.821	0.920	0.821
OCSVM [31]	0.039	0.195	0.559	0.119	0.512	0.905	0.512

Table S8. Evaluation of new critical variant detection (consider VOC/VOI as critical variants).

Model	MCC	F1_score	Precision	Recall	Accuracy	Specificity	AUC
AutoEncoder [34]	−0.014	0.004	0.002	0.078	0.839	0.842	0.460
ECOD [30]	−0.003	0.008	0.004	0.078	0.888	0.891	0.485
IForest [33]	−0.003	0.008	0.004	0.098	0.869	0.872	0.485
KNN [32]	0.105	0.035	0.018	0.852	0.794	0.793	0.822
LUNAR [35]	0.102	0.045	0.023	0.722	0.812	0.812	0.767
OCSVM [31]	0.008	0.010	0.005	0.207	0.875	0.878	0.543
AutoEncoder+AutoEncoder	−0.011	0.004	0.002	0.078	0.860	0.862	0.470
AutoEncoder+ECOD	−0.007	0.006	0.003	0.039	0.919	0.923	0.481
AutoEncoder+IForest	−0.010	0.004	0.002	0.078	0.866	0.869	0.474
AutoEncoder+KNN	−0.011	0.004	0.002	0.078	0.863	0.866	0.472
AutoEncoder+LUNAR	−0.011	0.004	0.002	0.078	0.861	0.864	0.471
AutoEncoder+OCSVM	−0.008	0.006	0.003	0.078	0.872	0.875	0.477
ECOD+AutoEncoder	0.000	0.008	0.004	0.078	0.907	0.910	0.494
ECOD+ECOD	−0.005	0.007	0.004	0.049	0.919	0.923	0.486
ECOD+IForest	0.000	0.008	0.004	0.078	0.908	0.911	0.495
ECOD+KNN	0.000	0.008	0.004	0.078	0.908	0.911	0.495
ECOD+LUNAR	0.000	0.008	0.004	0.078	0.907	0.910	0.494
ECOD+OCSVM	−0.003	0.008	0.004	0.078	0.894	0.897	0.488
IForest+AutoEncoder	−0.004	0.007	0.003	0.078	0.894	0.897	0.488
IForest+ECOD	−0.007	0.006	0.003	0.039	0.919	0.923	0.481
IForest+IForest	−0.002	0.007	0.004	0.078	0.902	0.905	0.492
IForest+KNN	0.010	0.012	0.006	0.127	0.902	0.905	0.516
IForest+LUNAR	0.024	0.015	0.008	0.225	0.899	0.901	0.563
IForest+OCSVM	−0.009	0.005	0.003	0.088	0.858	0.861	0.475
KNN+AutoEncoder	0.027	0.014	0.007	0.294	0.849	0.850	0.572
KNN+ECOD	−0.008	0.005	0.003	0.039	0.916	0.920	0.479
KNN+IForest	0.050	0.022	0.011	0.412	0.851	0.852	0.632
KNN+KNN	0.092	0.033	0.017	0.667	0.843	0.842	0.754
KNN+LUNAR	0.090	0.033	0.017	0.685	0.825	0.825	0.755
KNN+OCSVM	0.014	0.010	0.005	0.322	0.813	0.815	0.569
LUNAR+AutoEncoder	0.016	0.010	0.005	0.294	0.797	0.798	0.546
LUNAR+ECOD	−0.005	0.007	0.004	0.049	0.913	0.917	0.483
LUNAR+IForest	0.021	0.016	0.008	0.176	0.902	0.904	0.540
LUNAR+KNN	0.095	0.044	0.023	0.500	0.908	0.908	0.704
LUNAR+LUNAR	0.079	0.034	0.018	0.500	0.879	0.879	0.690
LUNAR+OCSVM	0.033	0.019	0.010	0.434	0.808	0.810	0.622
OCSVM+AutoEncoder	−0.005	0.006	0.003	0.059	0.909	0.913	0.486
OCSVM+ECOD	−0.005	0.007	0.004	0.049	0.919	0.923	0.486
OCSVM+IForest	−0.005	0.006	0.003	0.059	0.909	0.913	0.486
OCSVM+KNN	−0.005	0.006	0.003	0.059	0.911	0.914	0.486
OCSVM+LUNAR	−0.006	0.006	0.003	0.059	0.908	0.911	0.485

OCSVM+OCSVM	0.008	0.010	0.005	0.207	0.877	0.880	0.544

Table S9. Results of analog dynamic monitoring of new critical variant detection (consider VOC/VOI as critical variants).

country	date	Number of samples on the day	Number of samples during 30 days before the day	Real number of the new critical variants	KNN			LUNAR			KNN+LUNAR			KNN+KNN			LUNAR+KNN		
					Predicted number of the new critical variants	Recall	Specificity	Predicted number of the new critical variants	Recall	Specificity	Predicted number of the new critical variants	Recall	Specificity	Predicted number of the new critical variants	Recall	Specificity	Predicted number of the new critical variants	Recall	Specificity
Argentina	2021/10/1	18	708	0	5	/	0.722	5	/	0.722	5	/	0.722	5	/	0.722	6	/	0.667
	2021/10/2	16	675	0	3	/	0.813	1	/	0.938	4	/	0.750	4	/	0.750	2	/	0.875
	2021/10/3	5	654	0	2	/	0.600	3	/	0.400	2	/	0.600	2	/	0.600	2	/	0.600
	2021/10/4	23	621	1	5	1	0.818	2	1	0.955	4	1	0.864	4	1	0.864	3	1	0.909
	2021/10/5	19	627	0	0	/	1.000	3	/	0.842	1	/	0.947	1	/	0.947	1	/	0.947
	2021/10/6	25	621	0	3	/	0.880	3	/	0.880	3	/	0.880	3	/	0.880	3	/	0.880
	2021/10/7	31	600	0	1	/	0.968	1	/	0.968	2	/	0.935	2	/	0.935	1	/	0.968
	2021/10/8	22	593	1	3	0	0.857	2	0	0.905	2	0	0.905	2	0	0.905	1	0	0.952
	2021/10/9	13	595	0	0	/	1.000	0	/	1.000	1	/	0.923	1	/	0.923	0	/	1.000
	2021/10/10	20	589	0	1	/	0.950	9	/	0.550	1	/	0.950	1	/	0.950	0	/	1.000
	2021/10/11	18	590	0	1	/	0.944	5	/	0.722	1	/	0.944	1	/	0.944	1	/	0.944
	2021/10/12	55	595	0	2	/	0.964	2	/	0.964	2	/	0.964	2	/	0.964	2	/	0.964
	2021/10/13	35	638	0	2	/	0.943	1	/	0.971	1	/	0.971	1	/	0.971	1	/	0.971
	2021/10/14	51	648	0	4	/	0.922	3	/	0.941	4	/	0.922	4	/	0.922	3	/	0.941
	2021/10/15	47	674	0	4	/	0.915	3	/	0.936	4	/	0.915	4	/	0.915	3	/	0.936
	2021/10/16	31	695	0	1	/	0.968	1	/	0.968	1	/	0.968	1	/	0.968	2	/	0.935
	2021/10/1	12	694	0	0	/	1.000	0	/	1.000	0	/	1.000	0	/	1.000	0	/	1.000

	7																		
	2021/10/18	60	683	0	2	/	0.967	51	/	0.150	1	/	0.983	1	/	0.983	2	/	0.967
	2021/10/19	52	730	0	4	/	0.923	3	/	0.942	3	/	0.942	3	/	0.942	3	/	0.942
	2021/10/20	53	773	0	4	/	0.925	2	/	0.962	4	/	0.925	4	/	0.925	3	/	0.943
	2021/10/21	57	804	0	6	/	0.895	2	/	0.965	6	/	0.895	6	/	0.895	2	/	0.965
	2021/10/22	58	836	0	10	/	0.828	6	/	0.897	4	/	0.931	4	/	0.931	6	/	0.897
	2021/10/23	34	883	0	3	/	0.912	4	/	0.882	3	/	0.912	3	/	0.912	3	/	0.912
	2021/10/24	24	881	0	2	/	0.917	3	/	0.875	3	/	0.875	3	/	0.875	2	/	0.917
	2021/10/25	68	885	0	7	/	0.897	7	/	0.897	6	/	0.912	6	/	0.912	5	/	0.926
	2021/10/26	80	936	0	6	/	0.925	5	/	0.938	6	/	0.925	6	/	0.925	3	/	0.963
	2021/10/27	72	1005	1	3	1	0.972	3	1	0.972	5	1	0.944	4	1	0.958	2	1	0.986
	2021/10/28	77	1057	0	9	/	0.883	5	/	0.935	7	/	0.909	7	/	0.909	4	/	0.948
	2021/10/29	73	1115	0	10	/	0.863	13	/	0.822	15	/	0.795	11	/	0.849	9	/	0.877
	2021/10/30	30	1169	0	2	/	0.933	1	/	0.967	3	/	0.900	3	/	0.900	1	/	0.967
	2021/10/31	18	1179	0	0	/	1.000	1	/	0.944	0	/	1.000	0	/	1.000	1	/	0.944
China	2022/5/1	20	338	0	3	/	0.850	2	/	0.900	3	/	0.850	3	/	0.850	3	/	0.850
	2022/5/2	21	358	0	4	/	0.810	4	/	0.810	6	/	0.714	6	/	0.714	2	/	0.905
	2022/5/3	27	378	0	2	/	0.926	2	/	0.926	4	/	0.852	4	/	0.852	1	/	0.963
	2022/5/4	26	401	0	2	/	0.923	2	/	0.923	2	/	0.923	2	/	0.923	2	/	0.923

	2022/5/5	28	415	0	7	/	0.750	8	/	0.714	8	/	0.714	8	/	0.714	7	/	0.750
	2022/5/6	3	439	1	0	0	1.000	0	0	1.000	0	0	1.000	0	0	1.000	0	0	1.000
	2022/5/7	20	440	0	4	/	0.800	2	/	0.900	3	/	0.850	3	/	0.850	3	/	0.850
	2022/5/8	28	448	0	0	/	1.000	1	/	0.964	1	/	0.964	1	/	0.964	1	/	0.964
	2022/5/9	13	470	0	2	/	0.846	2	/	0.846	2	/	0.846	2	/	0.846	2	/	0.846
	2022/5/10	14	477	0	3	/	0.786	3	/	0.786	3	/	0.786	3	/	0.786	3	/	0.786
	2022/5/11	15	482	0	1	/	0.933	1	/	0.933	2	/	0.867	2	/	0.867	2	/	0.867
	2022/5/12	31	489	0	2	/	0.935	6	/	0.806	2	/	0.935	2	/	0.935	2	/	0.935
	2022/5/13	16	510	0	1	/	0.938	1	/	0.938	1	/	0.938	1	/	0.938	2	/	0.875
	2022/5/14	19	523	0	1	/	0.947	1	/	0.947	1	/	0.947	1	/	0.947	1	/	0.947
	2022/5/15	23	538	0	0	/	1.000	0	/	1.000	0	/	1.000	0	/	1.000	0	/	1.000
	2022/5/16	26	559	0	0	/	1.000	0	/	1.000	0	/	1.000	0	/	1.000	0	/	1.000
	2022/5/17	14	580	0	0	/	1.000	0	/	1.000	0	/	1.000	0	/	1.000	0	/	1.000
	2022/5/18	24	591	0	0	/	1.000	0	/	1.000	0	/	1.000	0	/	1.000	0	/	1.000
	2022/5/19	21	607	0	0	/	1.000	0	/	1.000	0	/	1.000	0	/	1.000	0	/	1.000
	2022/5/20	31	622	0	0	/	1.000	0	/	1.000	0	/	1.000	0	/	1.000	0	/	1.000
	2022/5/21	25	651	0	0	/	1.000	0	/	1.000	0	/	1.000	0	/	1.000	0	/	1.000
	2022/5/22	32	676	0	0	/	1.000	0	/	1.000	0	/	1.000	0	/	1.000	0	/	1.000
	2022/5/23	18	699	0	0	/	1.000	0	/	1.000	0	/	1.000	0	/	1.000	0	/	1.000
	2022/5/24	17	691	0	0	/	1.000	0	/	1.000	0	/	1.000	0	/	1.000	0	/	1.000
	2022/5/25	20	691	0	0	/	1.000	1	/	0.950	0	/	1.000	0	/	1.000	0	/	1.000
	2022/5/26	17	693	0	0	/	1.000	0	/	1.000	0	/	1.000	0	/	1.000	0	/	1.000
	2022/5/27	12	672	0	0	/	1.000	0	/	1.000	0	/	1.000	0	/	1.000	0	/	1.000
	2022/5/28	11	658	0	0	/	1.000	1	/	0.909	0	/	1.000	0	/	1.000	4	/	0.636
	2022/5/29	7	638	0	0	/	1.000	0	/	1.000	0	/	1.000	0	/	1.000	0	/	1.000
	2022/5/30	9	608	1	0	0	1.000	0	0	1.000	0	0	1.000	0	0	1.000	1	1	1.000
	2022/5/31	19	588	0	0	/	1.000	2	/	0.895	0	/	1.000	0	/	1.000	1	/	0.947
Portugal	2021/4/1	12	1010	0	5	/	0.583	8	/	0.333	4	/	0.667	5	/	0.583	5	/	0.583
	2021/4/2	8	911	0	1	/	0.875	1	/	0.875	1	/	0.875	1	/	0.875	1	/	0.875
	2021/4/3	5	798	0	0	/	1.000	0	/	1.000	0	/	1.000	0	/	1.000	0	/	1.000
	2021/4/4	8	710	0	1	/	0.875	1	/	0.875	1	/	0.875	1	/	0.875	1	/	0.875
	2021/4/5	233	616	1	51	0	0.780	49	1	0.793	48	0	0.793	51	0	0.780	41	0	0.823

	2021/4/6	196	790	1	12	0	0.938	9	0	0.954	13	0	0.933	13	0	0.933	9	0	0.954
	2021/4/7	166	942	0	36	/	0.783	31	/	0.813	38	/	0.771	38	/	0.771	33	/	0.801
	2021/4/8	189	1023	0	8	/	0.958	6	/	0.968	7	/	0.963	7	/	0.963	6	/	0.968
	2021/4/9	200	1154	0	12	/	0.940	9	/	0.955	13	/	0.935	13	/	0.935	10	/	0.950
	2021/4/10	126	1277	0	7	/	0.944	6	/	0.952	10	/	0.921	10	/	0.921	7	/	0.944
	2021/4/11	50	1331	0	1	/	0.980	2	/	0.960	1	/	0.980	1	/	0.980	3	/	0.940
	2021/4/12	78	1335	0	5	/	0.936	5	/	0.936	5	/	0.936	5	/	0.936	5	/	0.936
	2021/4/13	80	1364	0	5	/	0.938	13	/	0.838	4	/	0.950	5	/	0.938	5	/	0.938
	2021/4/14	63	1419	0	6	/	0.905	5	/	0.921	5	/	0.921	6	/	0.905	6	/	0.905
	2021/4/15	82	1469	0	4	/	0.951	24	/	0.707	3	/	0.963	3	/	0.963	3	/	0.963
	2021/4/16	60	1547	0	7	/	0.883	8	/	0.867	8	/	0.867	8	/	0.867	6	/	0.900
	2021/4/17	42	1600	0	0	/	1.000	0	/	1.000	0	/	1.000	0	/	1.000	0	/	1.000
	2021/4/18	21	1641	1	0	0	1.000	0	0	1.000	0	0	1.000	0	0	1.000	0	0	1.000
	2021/4/19	30	1657	0	3	/	0.900	3	/	0.900	3	/	0.900	3	/	0.900	3	/	0.900
	2021/4/20	5	1686	0	1	/	0.800	1	/	0.800	1	/	0.800	1	/	0.800	1	/	0.800
	2021/4/21	9	1691	1	2	1	0.875	2	1	0.875	2	1	0.875	2	1	0.875	2	1	0.875
	2021/4/22	2	1697	0	0	/	1.000	0	/	1.000	0	/	1.000	0	/	1.000	0	/	1.000
	2021/4/23	1	1696	0	0	/	1.000	1	/	0.000	0	/	1.000	0	/	1.000	0	/	1.000
	2021/4/24	6	1696	0	0	/	1.000	3	/	0.500	0	/	1.000	0	/	1.000	0	/	1.000
	2021/4/25	1	1696	0	1	/	0.000	1	/	0.000	1	/	0.000	1	/	0.000	1	/	0.000
	2021/4/26	4	1692	0	2	/	0.500	1	/	0.750	2	/	0.500	2	/	0.500	1	/	0.750
	2021/4/27	11	1695	0	0	/	1.000	0	/	1.000	1	/	0.909	1	/	0.909	1	/	0.909
	2021/4/28	3	1705	0	1	/	0.667	1	/	0.667	1	/	0.667	1	/	0.667	1	/	0.667
	2021/4/29	1	1704	0	1	/	0.000	1	/	0.000	1	/	0.000	1	/	0.000	1	/	0.000
	2021/4/30	8	1697	0	0	/	1.000	0	/	1.000	0	/	1.000	0	/	1.000	0	/	1.000

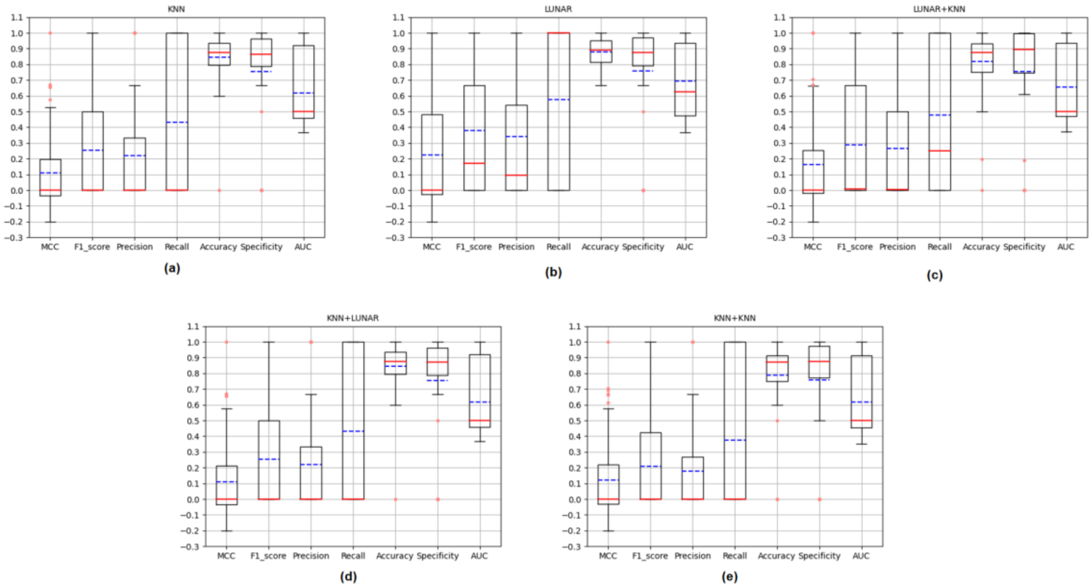

Figure S1. Compare the detection of all critical variants on the days they first appeared in three countries (consider VOC/VOI as critical variants). (**a**) The evaluation metrics of KNN; (**b**) the evaluation metrics of LUNAR; (**c**) the evaluation metrics of LUNAR+KNN; (**d**) the evaluation metrics of KNN+LUNAR; (**e**) the evaluation metrics of KNN+KNN. The red lines in the boxplots indicate the medians, the blue lines indicate the mean values, and the pink dots indicate the outliers.

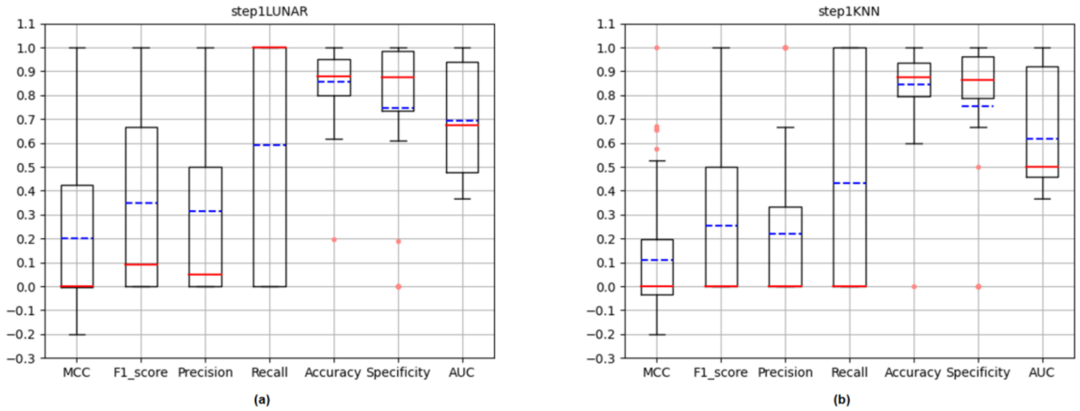

Figure S2. Compare the new variant detection of all critical variants on the days they first appeared in three countries (consider VOC/VOI as critical variants). (**a**) The evaluation metrics of LUNAR; (**b**) the evaluation metrics of KNN. The red lines in the boxplots indicate the medians, the blue lines indicate the mean values, and the pink dots indicate the outliers.

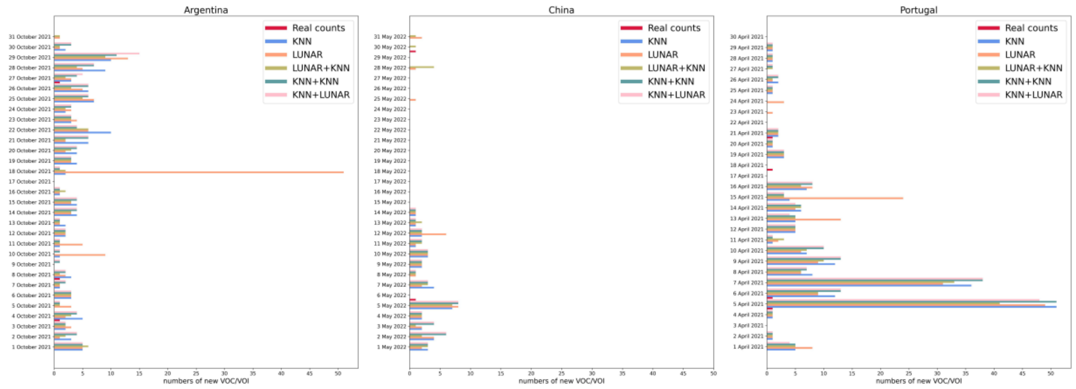

(a) (b) (c)

Figure S3. Analog dynamic monitoring of the new critical variants in three countries during a certain period (consider VOC/VOI as critical variants). (**a**) Comparison of the predicted quantity with the actual quantity in Argentina; (**b**) comparison of the predicted quantity with the actual quantity in China; (**c**) comparison of the predicted quantity with the actual quantity in Portugal

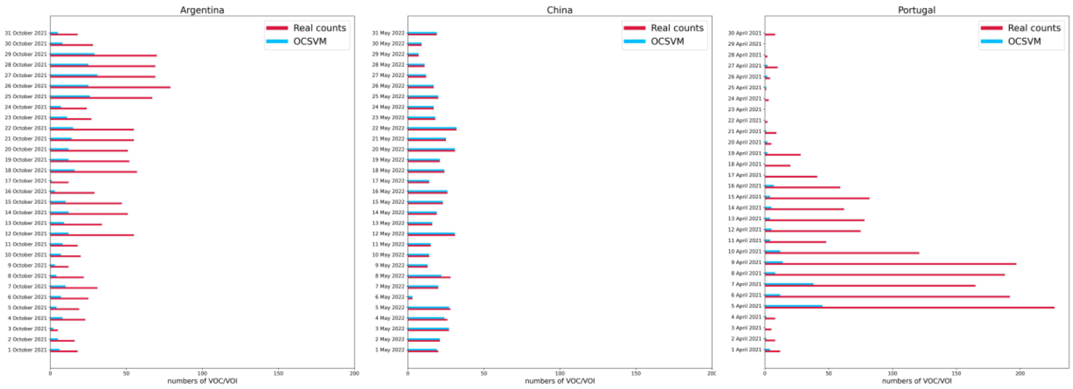

(a) (b) (c)

Figure S4. Analog dynamic monitoring of the critical variants in three countries during a certain period using OCSVM (consider VOC/VOI as critical variants). (**a**) Comparison of the predicted quantity with the actual quantity in Argentina; (**b**) comparison of the predicted quantity with the actual quantity in China; (**c**) comparison of the predicted quantity with the actual quantity in Portugal

References

30. Li, Z.; Zhao, Y.; Hu, X.; Botta, N.; Ionescu, C.; Chen, G. ECOD: Unsupervised Outlier Detection Using Empirical Cumulative Distribution Functions. *IEEE Trans. Knowl. Data Eng.* **2022**, *35*, 12181–12193. https://doi.org/10.1109/TKDE.2022.3159580.
31. Schölkopf, B.; Williamson, R.; Smola, A.; Shawe-Taylor, J.; Platt, J. Support Vector Method for Novelty Detection. In Proceedings of 12th International Conference on Neural Information Processing Systems, Denver, CO, USA, 29 November–4 December 1999; pp. 582–588.
32. Angiulli, F.; Pizzuti, C. Fast Outlier Detection in High Dimensional Spaces. In *Principles of Data Mining and Knowledge Discovery*; Springer: Berlin/Heidelberg, Germany, 2002; pp. 15–27.
33. Liu, F.T.; Ting, K.M.; Zhou, Z.-H. Isolation-Based Anomaly Detection. *ACM Trans. Knowl. Discov. Data* **2012**, *6*, 1–39. https://doi.org/10.1145/2133360.2133363.
34. Michelucci, U. Autoencoders. In *Applied Deep Learning with TensorFlow 2: Learn to Implement Advanced Deep Learning Techniques with Python*; Apress: Berkeley, CA, USA, 2022; pp. 257–283. https://doi.org/10.1007/978-1-4842-8020-1_9.
35. Goodge, A.; Hooi, B.; Ng, S.K.; Ng, W.S. LUNAR: Unifying Local Outlier Detection Methods via Graph Neural Networks. *AAAI Conf. Artif. Intell.* **2022**, *36*, 6737–6745.
